# Biggest of tinies: natural variation in seed size and mineral distribution in the ancient crop tef [*Eragrostis tef* (Zucc.) Trotter]

**DOI:** 10.3389/fpls.2024.1485819

**Published:** 2024-12-12

**Authors:** Eric D. Whisnant, Christian Keith, Louisa Smieska, Ju-Chen Chia, Abreham Bekele-Alemu, Olena K. Vatamaniuk, Robert VanBuren, Ayalew Ligaba-Osena

**Affiliations:** ^1^ Laboratory of Plant Molecular Biology and Biotechnology, Department of Biology, The University of North Carolina at Greensboro, Greensboro, NC, United States; ^2^ Cornell High Energy Synchrotron Source, Cornell University, Ithaca, NY, United States; ^3^ Plant Biology Section, School of Integrative Plant Science, Cornell University, Ithaca, NY, United States; ^4^ Department of Horticulture, Michigan State University, East Lansing, MI, United States

**Keywords:** *Eragrostis tef*, *Eragrostis pilosa*, seed size, mineral distribution, mineral concentration, seed size regulating genes

## Abstract

Tef [*Eragrostis tef* (Zucc.) Trotter] is the major staple crop for millions of people in Ethiopia and Eritrea and is believed to have been domesticated several thousand years ago. Tef has the smallest grains of all the cereals, which directly impacts its productivity and presents numerous challenges to its cultivation. In this study, we assessed the natural variation in seed size of 189 tef and 11 accessions of its wild progenitor Indian lovegrass (*Eragrostis pilosa* (L.) P. Beauv.) and explored the mineral distribution of representative accessions. Our findings revealed significant natural variation in seed size and mineral concentration among both the tef and *E. pilosa* accessions. We observed significant variation in seed length, seed width, and seed area among the accessions of both *Eragrostis* spp. we analyzed. Using representative accessions of both species, we also found significant variation in 1000-grain weight. The observed variation in seed size attributes prompted us to use comparative genomics to identify seed size regulating genes based on the well-studied and closely related monocot cereal rice [*Oryza sativa* (L.)]. Using this approach, we identified putative orthologous genes in the tef genome that belong to a number of key pathways known to regulate seed size in rice. Phylogenetic analysis of putative tef orthologs of ubiquitin-proteasome, G-protein, MAPK, and brassinosteroid (BR)-family genes indicate significant similarity to seed size regulating genes in rice and other cereals. Because tef is known to be more nutrient-dense than other more common cereals such as rice, wheat, and maize, we also studied the mineral concentration of selected accessions using ICP-OES and explored their distribution within the seeds using synchrotron-based X-ray fluorescence (SXRF) microscopy. The findings showed significant variation in seed mineral concentration and mineral distribution among the selected accessions of both *Eragrostis* spp. This study highlights the natural variation in seed size attributes, mineral concentration, and distribution, while establishing the basis for understanding the genetic mechanisms regulating these traits. We hope our findings will lead to a better understanding of the evolution of tef at the genetic level and for the development of elite tef cultivars to improve seed size, yield, and quality of the grains.

## 1 Introduction

Agricultural research has historically overlooked crops such as tef [*Eragrostis tef* (Zucc.) Trotter] which are often called “orphan crops” or “underutilized crops”, defined as crops that are regionally limited, indigenous, grown for subsistence farming, and often neglected scientifically (Talabi et al., 2022). Orphan crops typically lack the scale and size to compete with more globally popular crops. This means most agricultural research focuses on a small set of major crops. Orphan crops present a rich source of genetic diversity for highly nutritional foods, which are often limited in production to small areas of the world (Naylor et al., 2004; Talabi et al., 2022). Tef is an ancient cereal grain primarily grown in the Horn of Africa. Its exact period of domestication remains unclear but is believed to have occurred several thousand years ago, with estimates ranging from ~2000 to 8000 years ago (D’Andrea, 2008; Bultosa, 2016; Vavilov, 1951). Ethiopia is the center for its domestication, a hotspot for tef biodiversity, and the primary location of its production. Tef is Ethiopia’s major food crop, grown on over three million hectares of land by six million farmers (Tadele and Hibistu, 2021).

Tef is a self-pollinating allotetraploid C_4_ cereal (Assefa et al., 2015; Cheng et al., 2017) in the Chloridoideae subfamily of the Poaceae (grass family) (Cannarozzi et al., 2014; VanBuren et al., 2020). Unlike wheat, barley, and rice, tef grows efficiently in hot and arid climates and has natural resistance to many biotic and abiotic stresses (Bekele-Alemu and Ligaba-Osena, 2023; Girija et al., 2022). Tef is the only *Eragrostis* species that is actively cultivated of the 350 species in the *Eragrostis* genus (Bultosa and Taylor, 2004).

The evolution of tef from its wild progenitors has been under speculation for many years. Based on morphological, cytological, biochemical, and genomic data, it is believed that *Eragrostis pilosa* (L.) P. Beauv. was an original progenitor of tef (Jones et al., 1978; Bekele and Lester, 1981; Ingram and Doyle, 2003). Additionally, *E. pilosa* is the only species with which tef successfully crosses among the *Eragrostis* species and is known to have several useful traits including wider environmental adaptation, lodging tolerance, and early maturity (Ayele et al., 1999; Tefera et al., 2003; Ingram and Doyle, 2003). Their ancestral relationship has been reasserted by genotyping by sequencing (Kebede et al., 2018). This makes *E. pilosa* an interesting species for the introgression of agronomically desirable traits into tef (Talabi et al., 2022). Reintroducing traits from wild progenitors back into domesticated species has been shown to improve desirable traits in maize, lentils, and wheat (Zhang et al., 2023; Rajpal et al., 2023; Keilwagen et al., 2022).

Tef is often touted for its nutritional superiority compared to other more popular cereals such as wheat, rice, and maize. It is gluten-free, becoming an important source of food for people with gluten intolerances and Celiac disease (Spaenij-Dekking et al., 2005). Tef has a low glycemic index, which can be valuable for those with diabetes (Habte et al., 2022). Tef grains also contain 9.4–13.3% protein with an excellent balance of essential amino acids present (Bultosa and Taylor, 2004). Additionally, studies have shown tef to have antioxidant activity *in vitro* (Kotásková et al., 2016; Shumoy et al., 2017) and through mammalian cell-based studies (Cotter et al., 2023), adding to the list of characteristics that make tef a nutritionally important crop. Tef grains have been shown to have higher mineral content than other cereals crops. More specifically, tef has a greater iron concentration than other cereals including wheat, barley, rice, and sorghum (Mohammed et al., 2009; Gebru et al., 2020). Dietary iron is integral as a micronutrient for preventing and treating iron-deficiency anemia. Ligaba-Osena et al. (2021) showed that tef grains have more bioavailable iron in cell-based assays versus rice and wheat. This same study showed that tef outranked other sampled cereals in essential minerals including Fe, Ca, S, K, Mg, P, Mn, and Zn concentrations. Others have shown similar findings when analyzing the mineral content in tef grains (Nyachoti et al., 2021; Habte et al., 2022), highlighting the value of the grains for dietary supplementation of key macro- and micronutrients. Moreover, using elite cultivars grown in Ethiopia, Ereful et al. (2022) reported exceptionally prominent levels of the key micronutrients Fe and Zn and began to establish the genetic basis associated with these traits. Therefore, it is important to keep the nutritional quality of the grain into consideration for those interested in developing elite tef varieties.

Despite the nutritional advantages of tef consumption, its global consumption has been thwarted due to challenges associated with its cultivation and low yield. Attempts to breed high yielding varieties of tef through modern techniques have been hindered due to the paucity of research on tef globally. Moreover, molecular breeding techniques are less developed in tef due to a lack of transformation and regeneration methods. Recently, the use of morphogenic regulator genes has shown promise in tef trait improvement (Beyene et al., 2022), yet this technology remains to be optimized for wide application. There remain many limitations to tef cultivation. For example, tef is prone to shattering and lodging, which directly diminishes the yield. Lodging occurs when the stalk prematurely breaks or bends and is estimated to decrease the yield by 30-35%. This has prompted molecular breeding research to develop lodging-resistant varieties (Ben-Zeev et al., 2020). Jöst et al. (2015) identified semi-dwarf varieties of tef via a mutation in the *α – tubulin 1* gene, which improved tolerance to lodging. Beyene et al. (2022) have recently developed lodging tolerant semi-dwarf tef lines via CRISPR/Cas9 genome editing. Ligaba-Osena et al. (2020) previously found that supplementation with silicon (Si) improved overall plant performance and grain yield. Tef research has been lagging due to a lack of awareness from the scientific community and funding agencies. However, there is an increasing interest in tef research, due in part to its increase in global popularity for its nutritious and gluten-free grains, and the quality of its straw for animal feed (Miller, 2009; Davison et al., 2011; Anderson and Volesky, 2012).

Furthermore, tef grains are the smallest of all cereals, estimated to be only about 1/150^th^ the size of a wheat grain (Bultosa and Taylor, 2004). It is believed that tef’s seed size directly limits the harvest yield. Additionally, the small seed size presents challenges during seed sowing in the field, which can lead to poor population control and uneven distribution (Mengie et al., 2021). Overcrowding of crops leads to competition for light, water, and nutrients. This has been shown to diminish biomass and grain yield in tef (Mengie et al., 2021). Due to their small size, tef seeds are easily lost during harvest, which results in diminished yield. Unlike cereals with larger grains, tef cannot be mechanically harvested using standard equipment. Tef farmers must either harvest the grains by hand, or build/purchase specialty equipment, which is often expensive and adds an additional layer of difficulty to tef cultivation. Traditional breeding methods have been unsuccessful for breeding larger grain size in tef, which has necessitated the improvement of seed size via molecular breeding.

Seed development is an extraordinarily complex and multi-pathway process which is under the control of many transcription factors and hormones (Su et al., 2021; Alam et al., 2022). Thus, developing methodologies for the manipulation of seed development processes is difficult and complex. In many cereals, such as rice, large seed size is an economically important attribute which is used as a measure for yield and quality (Li et al., 2022). For example, large seed size has been associated with improved yield and germination in durum wheat (Akinci et al., 2008). Understanding the genetic mechanisms regulating seed size is a major area of research, in the hopes of developing higher yield varieties of crops. However, a comprehensive understanding of the mechanisms regulating seed size is not totally understood. Abiotic factors likely also affect seed development, leading to alterations in seed size and weight (Ma et al., 2023). The advancement of high-throughput sequencing technology has paved the way for robust genomic analysis. Such analyses interested in seed size regulation have shown potential for identifying genes involved in seed size regulation in *Arabidopsis*, rice, wheat, maize, and soybean (Alam et al., 2022).

In rice, seed size is almost entirely determined by the size of the hull (Alam et al., 2022). However, final seed size is regulated by many different regulatory pathways. The current models of seed size development suggest six essential pathways that regulate seed size: 1) ubiquitin-proteasome signaling, 2) mitogen activated protein kinase (MAPK) signaling, 3) haiku (IKU) signaling 4) guanine nucleotide-binding proteins (G-protein) signaling, 5) transcription factors, and 6) phytohormone signaling (Alam et al., 2022; Li et al., 2022; Li and Li, 2016). Broadly, each of these pathways have been shown to influence final grain length, width, and mass. Simplifying further, there are two overarching phases in seed development. First, seed morphogenesis is marked by cell proliferation, embryo development, endosperm development, and formation of the cotyledon. Second, the seed undergoes maturation of the embryo and enters into a dehydrated dormant state (Alam et al., 2022; Badoni et al., 2023; Mohapatra and Sahu, 2022). Seed development at the genetic level is extraordinarily complex, but better understood only in a handful of species.

Here we report the seed size phenotypes of 189 genetically unique tef and 11 *E. pilosa* accessions. Even among the smallest grains in the world, we have identified significant phenotypic variation in seed size attributes among most of the tef population. Additionally, we have begun to establish the possible genetic mechanisms regulating seed size in tef via comparative genomics, using sequences from rice (*Oryza sativa*) to generate putative orthologous coding sequences from the tef genome. Overall, we explored the natural variation in seed size in tef, as well as seed mineral concentration and localization in the grains, and attempted to establish an understanding of the genetic mechanisms potentially regulating seed size in tef. To our knowledge, this is the first study to quantify natural variation in seed size, map and quantify mineral distribution, and identify putative seed size regulating genes in an *Eragrostis* species.

## 2 Methodology

### 2.1 Seed images, measurements, and analysis

**Figure 1:**
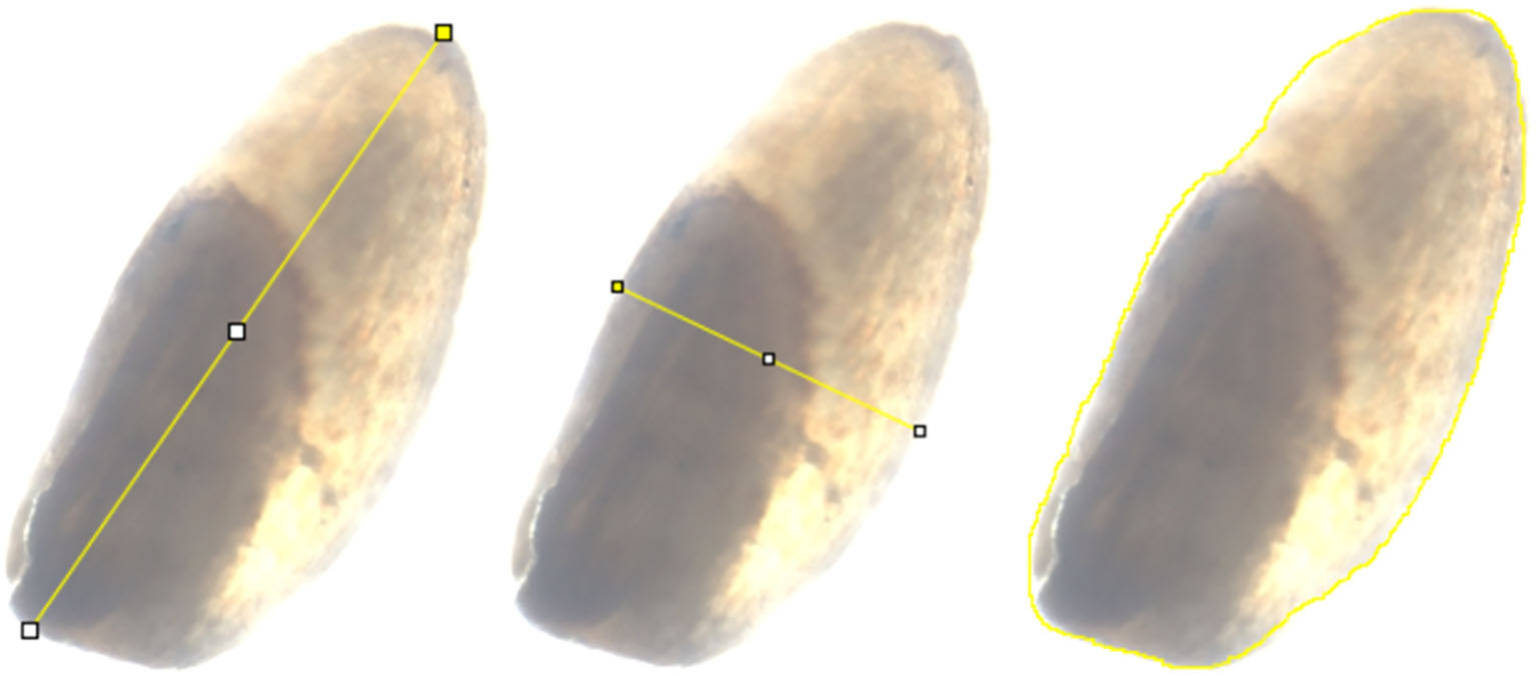
An example of how seed size attributes were measured in ImageJ. Images from left to right show measurement of seed length, seed width, and seed area. These measurements were converted to µm for seed length and seed width, and µm^2^ for seed area.


Bulked seeds of the tef diversity panel grown under field conditions were obtained from the USDA-ARS National Plant Germplasm System, Plant Germplasm Introduction and Testing Research Unit (Pullman, Washington, USA). The seeds were placed on a 76mm x 25mm concavity slide and imaged using a KEYENCE BZ-X710 All-in-one Fluorescence Microscope (Keyence Corporation of America, Ithica, Il) using the setting in **Supplementary Table S1**. ImageJ software v1.54g (Schneider et al., 2012) was used to determine seed size attributes including seed length, seed width, and seed area of 189 tef and 11 *E. pilosa* accessions. The length measurements were taken from the longest portion of the seed and the width measurements were taken from the widest portion of the seed, while the seed area corresponds to the region within the perimeter of the image (**Figure 1**). For each of the 189 tef and 11 *E. pilosa* accessions, ten seeds (replicates) were measured for length, width, and area. Each seed image included a 1000 µm scale bar. Ten replicate measurements of the scale bar were taken and averaged to generate a calibration factor from pixels to µm (seed length and width; Equations 1 and 2) or pixels^2^ to µm^2^ (area; Equation 3); the calibration factor is assigned pixels_c_ in Equations 1–3. According to the user guide (https://imagej.net/imaging/spatial-calibration), ImageJ takes area measurements as a count of pixels^2^ and straight-line measurements in pixels. We maintained the same microscope settings to acquire all the images. We found that after converting the seed measurement to a standardized unit (µm or µm^2^), the initial parameters were not as important, as long as the calibration was properly set.

(1) $$Width\;\left(µm\right)=pixels_{w}\;\times \;\frac{1000µm}{pixels_{c}}$$

(2) $$Length\;\left(µm\right)=pixels_{l}\;\times \;\frac{1000µm}{pixels_{c}}$$

(3) $$Area\;\left(µm^{2}\right)=pixels_{a}^{2}\;\times \;{\left(\frac{1000µm}{\;pixels_{c}}\right)}^{2}$$

Additionally, we also determined 1000-grain weight of representative tef accessions selected based on seed area analysis, and those accessions that are considered reference cultivars in tef research. For each accession, one thousand seeds were counted manually, and the mass was measured. This was conducted in quadruplicate and then averaged. Some common tef accessions used in this study include Dabi, a brown cultivar with medium-sized seeds and a reference cultivar; Magna, an ivory (white) variety with high consumer preference and relatively large seeds; and Dessie, another common, brown-colored cultivar widely grown in the U.S. Dabi, Magna, and Dessie have retained their common name, whereas other accessions are labeled with their Plant Introduction (PI) number **(Supplementary File 1)**.

### 2.2 Plant growth conditions tef seed mineral analysis

Seeds of selected *E. tef* accessions varying in seed size (small, medium, and large) along with the reference cultivar (Dabi) and the cultivar commonly grown in U.S. (Dessie), were grown under greenhouse conditions with supplemental light at constant temperature (28°C). Twenty seeds of each accession was planted in 2 L pots containing Sun Gro professional Mix (Sun Gro Horticulture, Agawam, MA). Plants were supplied with Osmocote^®^ Smart-release^®^ controlled-release fertilizer (Scotts Company, LLC, Marysville, OH) following the manufacturer’s recommended application rates. Seedlings were thinned down to five. Plants were supplemented with 1 L of 4.74 g/L Miracle-grow fertilizer (Scotts Miracle-Gro Co., Marysville, OH) solution (24–8–16) per flat containing two pots, and 1 L of 250 mg/L M.O.S.T. soluble trace elements (JR Peters, Allentown, PA) per flat every four weeks. Selected *E. pilosa* accessions were grown under the same conditions. All accessions were replicated four times. Seeds of plants grown in each pot were bulked at harvest, dried and processed for mineral analysis.

### 2.3 Seed mineral analysis

A total of 500 mg of ground seed was digested using concentrated HNO_3_ for 30 min in a microwave at 200°C, and mineral content was analyzed using Inductively Coupled Plasma Optical Emission Spectroscopy (ICP-OES).

### 2.4 Synchrotron x-ray fluorescence imaging

The procedures for synchrotron x-ray fluorescence (SXRF) imaging were described previously with slight modifications (Chia et al., 2023). Briefly, the seeds were placed between a layer of metal-free Kapton film and Kapton tape before being mounted onto 35-mm slide frames. The spatial distribution of elements was imaged via SXRF microscopy at the Functional Materials Beamline (FMB) of the Cornell High Energy Synchrotron Source (CHESS). FMB employs an undulator source and an energy of 9.7 keV was selected using a side-bounce monochromator (Smieska et al., 2023; Stoupin et al., 2021). The beam was focused to 3 µm tall x 15 µm wide containing approximately 3e10 ph/s using a set of compound refractive lenses (RXoptics, Monschau, Germany). SXRF signal was collected in flyscan mode along the vertical axis with a pixel size of 20 µm and a dwell time of 80 ms, using a Vortex ME4 detector (Hitachi, Japan) and an Xspress3 signal processor (Quantum Detectors, UK). SXRF peak areas were fit in Praxes software (https://github.com/praxes/praxes) which is based on PyMCA (http://dx.doi.org/10.1016/j.sab.2006.12.002). Thin foil calibration standards (Micromatter, Canada) were used to obtain a flux to concentration conversion and provide area densities in units of µg/cm^2^.

### 2.5 Comparative genomics

A list of seed development genes in rice was compiled in a review by Li et al. (2022) and Li and Li (2016). We used these lists as a reference to identify genes in rice that are specifically implicated in controlling grain size. Rice gene nucleotide coding sequences (CDS) were generated by searching the Gene ID number in NCBI (National Center for Biotechnology Information) database (https://www.ncbi.nlm.nih.gov/); Search criteria: All Databases). Using the rice CDS sequences as query, we searched the most similar CDS from wheat (*Triticum aestivum*; taxid: 4565), maize (*Zea mays*; taxid: 4577), barley (*Hordeum vulgare* L.; taxid: 4513), sorghum (*Sorghum bicolor* (L.) Moench; taxid: 4558) and *Arabidopsis* (*Arabidopsis thaliana* (L.) Heynh.; taxid: 3702). We also included rice (*Oryza sativa* (japonica cultivar group); taxid: 39947) in this search as a control comparison to ensure the correctness of the query sequence. Then, the tef CDSs were gathered using CoGeBlast, using the rice gene as the reference sequence (https://genomevolution.org/coge/CoGeBlast.pl). Rice CDS nucleotide sequences were BLAST against the tef genome (Selected Genome: *Eragrostis tef* (tef) id 50954 PacBio unmasked vV3; Maker, PacBio: (id 50954) vV3 unmasked 577,738,711nt). Rice sequences were used as the reference in all but two instances, where the *Sorghum bicolor* sequence was used to derive the *RGG1* and *AGO17* orthologs from tef. To simplify the phylogenetic analysis, we selected a single allele with the highest predicted similarity. However, since tef contains two sub-genomes, we have included both alleles of each putative seed size regulating gene in **Supplementary File 2**. The BLAST parameters were set at an E-Value cutoff of 1e-30, with other parameters left standard. This cutoff was set to ensure the tef sequences we acquired could be classified as putative homologs or orthologs using the standard cutoff for determining homology using BLAST (*E< 1e-5*) (Choudhuri, 2014), or the cutoff others have indicated for added level of scrutiny for nucleotide comparisons (*E< 1e-10*) (Pearson, 2013).

Gene phylogenies were grouped by their common regulatory pathway or functional category. Sequences were aligned using MUSCLE and the gene phylogenies constructed using a Maximum Likelihood tree building algorithm in MEGA v11.0.13 (Tamura et al., 2021). The trees were converted to Newick file format and uploaded to Interactive Tree of Life (ITOL) for final formatting and annotation (https://itol.embl.de/) (Letunic and Bork, 2024). The Newick files are included as **Supplementary Data**.

Functional analysis of rice genes was conducted using ShinyGO v0.80 (http://bioinformatics.sdstate.edu/go/), which was used to identify key biological processes involved in seed size regulation in rice (Ge et al., 2020). Our list consisted of reported seed size regulating genes that have been cloned and characterized in rice, compared against the rice genome background (Oryza sativa Japonica Group genes IRGSP-1.0; TaxID: 39947). Genes were clustered by GO Biological Processes (**Supplementary File 3**), and extracted using FDR (false discovery rate; *FDR< 0.05*) and enrichment score. ShinyGO defines Fold Enrichment as the percentage of genes in the list belonging to a pathway, divided by the corresponding percentage in the background (Ge et al., 2020; http://bioinformatics.sdstate.edu/go74/#:~:text=Fold%20Enrichment%20is%20defined%20as,a%20certain%20pathway%20is%20overrepresented). Quoted from the ShinyGO v.80 website, “FDR refers to how likely the enrichment is by chance; Fold Enrichment indicates how drastically genes of a certain pathway is overrepresented.” The purpose of this analysis was to highlight the pathways which are important to the regulation of seed size in rice. For those focused on tef breeding, genes of those pathways may be a key starting place for gene manipulation in tef, bearing the mechanisms regulating seed size in both cereals are similar.

### 2.6 Data analysis

Analysis of seed size attributes and ICP-OES results was performed in SAS Enterprise Guide v8.3.8.206 (SAS Institute Inc., Cary, NC USA). We conducted one-way analysis of variance (ANOVA) to determine if there was significant difference in mean seed width, seed length, and seed area among the 189 tef and 11 *E. pilosa* accessions. For the tef accessions, we binned accessions from each seed size attribute as small, medium, or large, and selected 10 accessions from each group for simplicity. These represent the 10 largest, medium, and smallest accessions for seed area, length, or width. For seed length, seed width, and seed area of the 30 representative accessions, we performed another one-way ANOVA, followed by a Student-Newman-Keuls (SNK) *post-hoc* test for means comparison. Lastly, ICP-OES results were averaged and analyzed by one-way ANOVA, followed by a SNK *post-hoc* test. Figures were generated using GraphPad Prism v10.0.3 (GraphPad software, Boston, Massachusetts, USA).

## 3 Results

### 3.1 Natural variation in seed size of *E. tef* and *E. pilosa* accessions

In this study, we analyzed the natural variation in seed size of 189 tef and 11 *E. pilosa* genotypes. Seed images were acquired by microscopy and seeds size attributes, including seed length, width, and area was measured. Additionally, 1000-grain weight was generated for selected tef and *E. pilosa* accessions.

**Figure 2:**
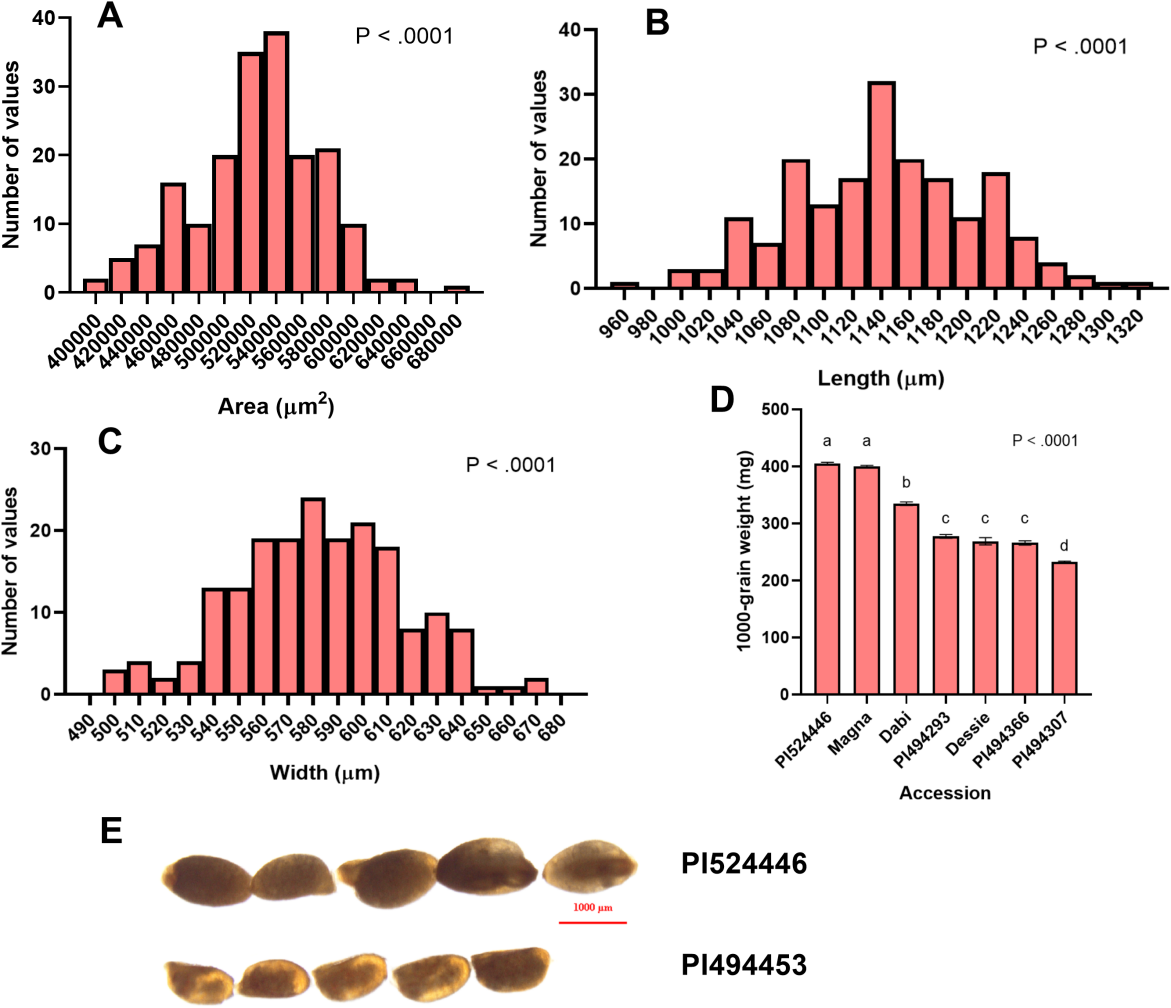
Measurements of tef seed area, length, width, and 1000-grain weight. **(A)** Frequency distribution of tef mean seed area (µm^2^), **(B)** Seed length (µm), **(C)** Seed width (µm), and **(D)** 1000-grain weight for representative tef varieties. Ten replicate measurements (n = 10) of seed length, seed width, and seed area were taken and averaged for each accession. Four replicates (n = 4) were weighed and averaged to generate the 1000-grain weight. Data was analyzed by one-way ANOVA and mean comparison using the Student-Newman-Keuls (SNK) *post-hoc* test. One-way ANOVA found that differences between accessions for all seed size attributes was statistically significant (*p< 0.0001*). Bars bearing the same letter are not statistically significant (*p< 0.05*). **(E)** Visual comparison of accessions with large (PI524446) and small (PI494453) seeds. Image was acquired under 1x magnification using a Nikon SMZ18 Stereoscope (Nikon, Kanagawa, Japan).

**Figure 3:**
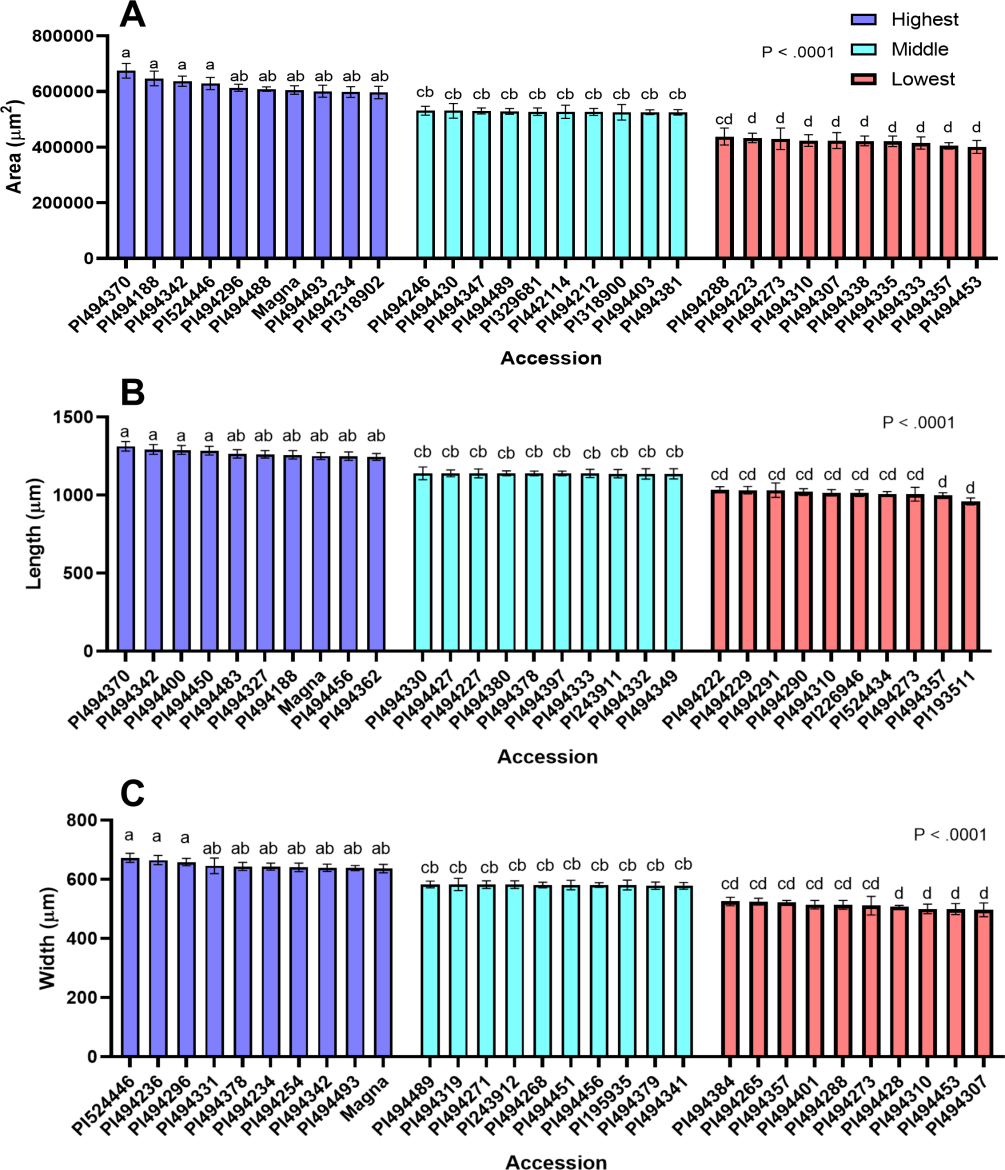
Seed size attributes of 30 representative tef accessions. Accessions were binned as high, medium, or low for seed area, length, and width. The data of 10 accessions representing the highest, medium, and lowest categories (30 total) are presented for mean seed area **(A)**, length **(B)** and width **(C)**. Ten replicate measurements (n = 10) of seed length, seed width, and seed area were taken and averaged for each accession. The 30 accessions were analyzed by one-way ANOVA. One-way ANOVA found that differences between accessions for all seed size attributes was statistically significant (*p<0.0001*). Statistical mean comparison used the SNK *post-hoc* test. Bars bearing the same letter are not significantly different (*p< 0.05*).

The distribution of seed areas of sampled tef accessions is normal (x̄ = 525,565 µm^2^; sd = 49,102 µm^2^) but slightly left skewed (**Supplementary Table S2**; **Figure 2A**). The center half (interquartile range, IQR) of the tef accessions fall within a range of 498,457 µm^2^ and 555,9739 µm^2^, with the largest and smallest accessions being nearly 25% larger and 27% smaller than the mean, respectively. One-way ANOVA revealed a statistically significant difference (*p< 0.0001*) between the 189 tef varieties in mean seed area. Seed areas of accessions PI494370 and PI494453 were identified as the largest and smallest, respectively (**Figure 3A**). Seeds of PI494370 are about 51% larger than the smallest accession PI494453. To better represent our data, we selected ten accessions with the largest, medium, and smallest areas (30 in total). One-way ANOVA revealed that the difference in seed area among the 30 selected accessions is statistically significant (**Figure 3A**). A *post-hoc* test indicated that the ten largest accessions were significantly larger than the ten smallest. However, no significant difference was found between the medium ten accessions and six of the larger and one of the smaller tef accessions.

The distribution of tef accessions by seed length is normal (x̄ = 1142 µm; sd = 65.75 µm), and slightly left skewed, with the IQR of the accessions falling between 1095µm and 1185µm (**Figure 2B**). The 189 tef accessions were significantly different (*p< 0.0001*) in seed length. The longest accession was identified as PI494370, which also has the largest seed area. The seed length of PI494370 is 31% larger than PI193511, the shortest accession (**Figure 3B**), and were found to be significantly different in the 30-accession comparison (*p< 0.05*; **Figure 3B**).

Like seed area and length, the distribution of accessions by seed width is normal (x̄ = 582.4 µm; sd = 33.98 µm) and slightly left skewed. The IQR falls within 559.1 µm and 605.7 µm. One-way ANOVA showed a statistically significant difference in seed width for the 189 tef accessions (*p< 0.0001*; **Figure 2C**). The seed width of accessions PI524446 and PI494307 were the widest and most narrow, respectively, among the 189 tef accessions (**Figure 3C**). Seed of PI524446 are 30% wider than that of PI494307. One-way ANOVA of the 30-accession comparison of seed width revealed significant differences among the accessions. A *post-hoc* test indicated significant differences between the ten widest and ten most narrow accessions (**Figure 3C**). No significant difference was found between the ten medium width accessions and seven widest and six most narrow accessions. A summary of these findings are in **Supplementary Table S2**.

To determine the 1000-grain weight we counted 1000 seeds of seven tef accessions representing large, medium, and small seeds, and took the weights of the seeds. PI524446, which also had the widest seeds, had the highest 1000-grain weight, while PI494307 was found to have the lowest 1000-grain weight. The latter also has the smallest seed width. The 1000-grain weight of PI524446 was 1.54 times higher than that of PI494307, whose difference was statistically significant (**Figure 2D**).

**Figure 4:**
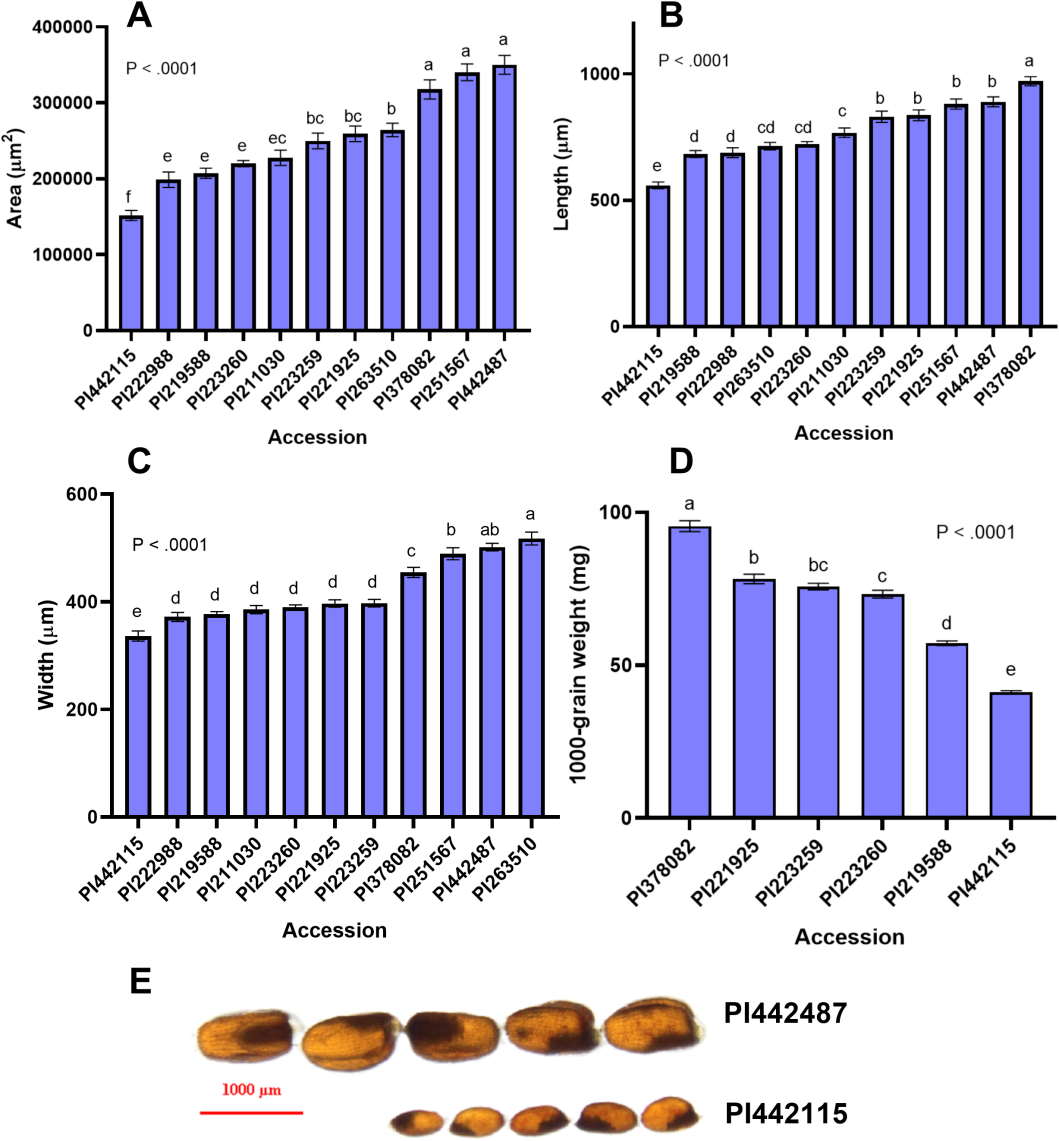
Seed size attributes of *E. pilosa* **(A)** Seed area (µm^2^), **(B)** Seed length (µm) **(C)** Seed width (µm), and **(D)** 1000-grain weight for representative *E. pilosa* varieties. Ten replicate measurements (n = 10) of seed length, width, and area were taken and averaged for each accession. Four replicates (n = 4) were taken and averaged to generate the 1000-grain weight. Data were analyzed by one-way ANOVA. One-way ANOVA found that differences between accessions for all seed size attributes was statistically significant (*p<0.0001*). The SNK *post-hoc* test was used for means comparison. Bars bearing different letters are significantly different (*p< 0.05*). **(E)** Visual comparison of accessions with the largest (PI442487) and smallest (PI442115) seeds by area. Seeds images were acquired under 1x magnification, taken on a Nikon SMZ18 Stereoscope (Nikon, Kanagawa, Japan).

**Figure 5:**
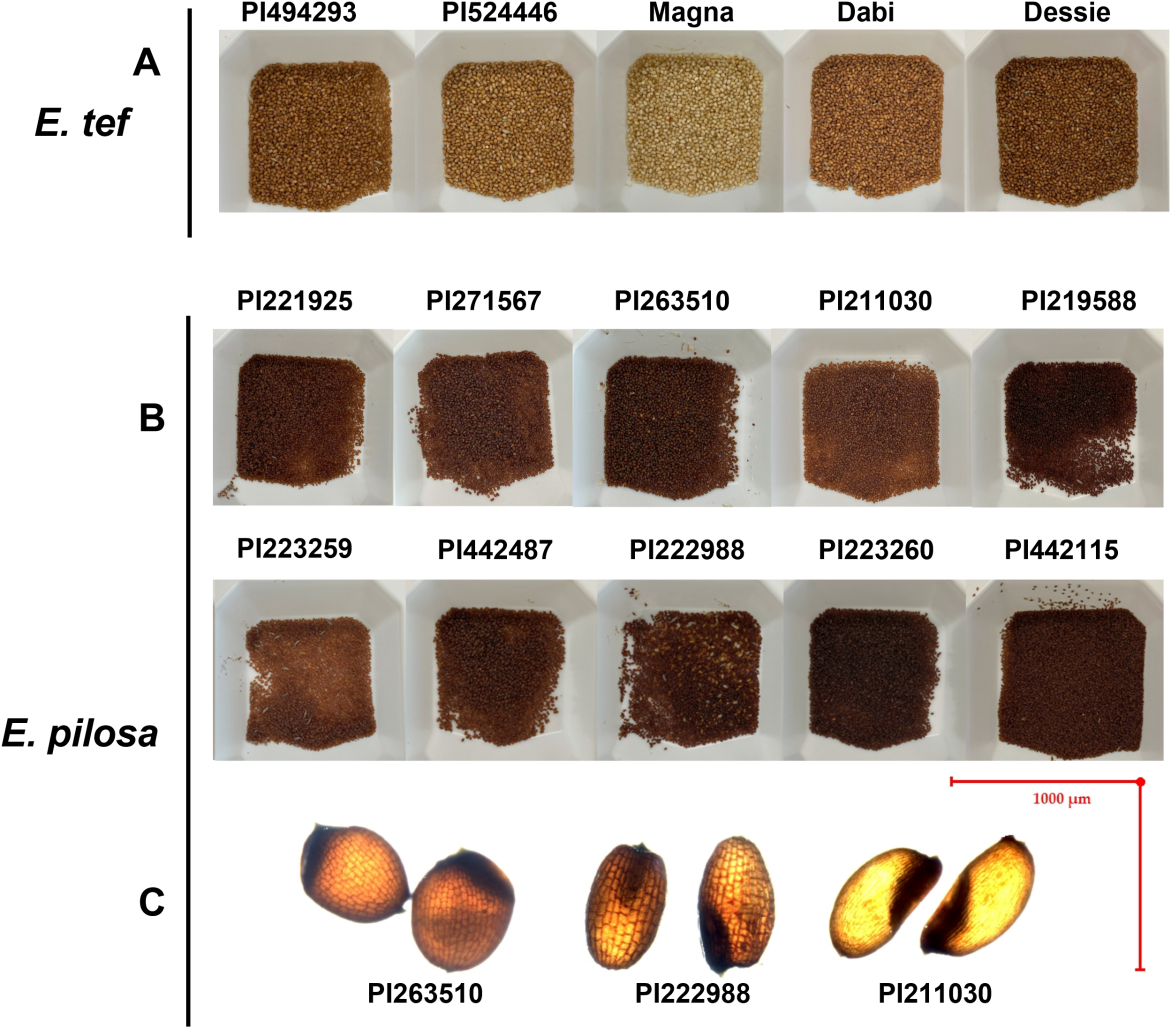
Comparison of selected **(A)** tef and **(B)** *E. pilosa* genotypes. **(C)** Images highlight phenotypes observed from *E. pilosa*, including the reticulate-patterned seed coat observed in a few accessions (e.g., PI222988) compared to the smooth seed coat in others (e.g., PI211030). A diminished length-to-width ratio was observed in PI263510. We observed differences in seed shape in some *E. pilosa* accessions, including more oblong seeds with emarginated apices (e.g., PI222988) in comparison to the more ovular seed shape of PI211030.

During the course of the seed size measurement and analysis, we identified a unique accession PI442115, which was substantially smaller than the tef accessions in our sample. PI442115 was a total outlier in seed size and color compared to the other tef accessions. PI442115 more closely resembled *E. pilosa* in color and in size than it did to tef (**Figure 4**). It was significantly smaller and much darker in color (**Figure 5**). This accession was mischaracterized in the USDA-ARS Germplasm Resource Information Network (GRIN) database as tef. This information prompted us to expand the study to include all the *E. pilosa* accessions available at the U.S. National Plant Germplasm Center.

We also measured seed length, width, and area for 11 *E. pilosa* accessions. Our analysis revealed significant variation among the seed size attributes of *E. pilosa.* Seed area of accession PI442487 (350,174 µm^2^) was 1.79 times that of PI442115 (151,744µm^2^). PI378082 has the longest seeds, and PI442115 has the shortest seeds among the *E. pilosa* accessions. The widest *E. pilosa* accession, PI263510, was 42% wider than PI442115. The 1000-grain weight of PI378082 is twice that of PI442115. Seed size attributes (area, length, width, and 1000-grain weight) of PI442115 are the smallest of all the tef and *E. pilosa* accessions we analyzed. A summary of these findings is in **Supplementary Table S3**. The analysis of *E. pilosa* grain length, width, area, and 1000-grain weight revealed significant variation in seed size among all of the available genotypes. We suspect there is a genetic basis behind this natural variation, but this remains to be validated. Furthermore, there is the potential for identifying alleles from tef’s progenitor species that can shed light onto the evolution of tef domestication or can be utilized as a source of genes for introgression of agronomically desirable traits into tef. In rice, a handful of alleles regulating seed size have been identified in a similar way, using wild rice species such as *Oryza rufipogon*, the *O. sativa* progenitor, and *O. barthii*, the progenitor of African rice *O. glaberrima*, to identify alleles that were lost or selected for during domestication (Jiang et al., 2019; Luo et al., 2013; Wu et al., 2017).

Additionally, we identified morphological differences in both the tef and *E. pilosa* seeds. Most *E. pilosa* grains appear distinctly darker than tef grains. In comparison, tef grains have a lighter colored seed coat, with a smooth outer surface. Some of the *E. pilosa* grains showed clear darkened reticulate patterned seed coat (For an example: **Figure 5C**; PI222988). This was also reported by Kreichstitz et al. (2009). None of the tef accessions we sampled expressed this type of patterning. Interestingly, not all of the *E. pilosa* accessions showed reticulate seed coat patterning but retained the darker seed coat coloration. Specifically, PI211030, PI221925, and PI223259 have fingerprint-like patterning and more closely resemble tef grains in this aspect (For an example: **Figure 5C**; PI211030). Additionally, the non-reticulate *E. pilosa* accessions are more ovular (**Figure 5C**; PI211030), whereas the reticulate patterned seeds are oblong, with more pronounced emarginated apices (**Figure 5C**; PI222988). The seeds from PI263510 (*E. pilosa*) appeared to be more spherical, with a highly diminished length-to- width ratio (**Figure 5C**) (data not shown).

### 3.2 Mineral analysis

**Figure 6:**
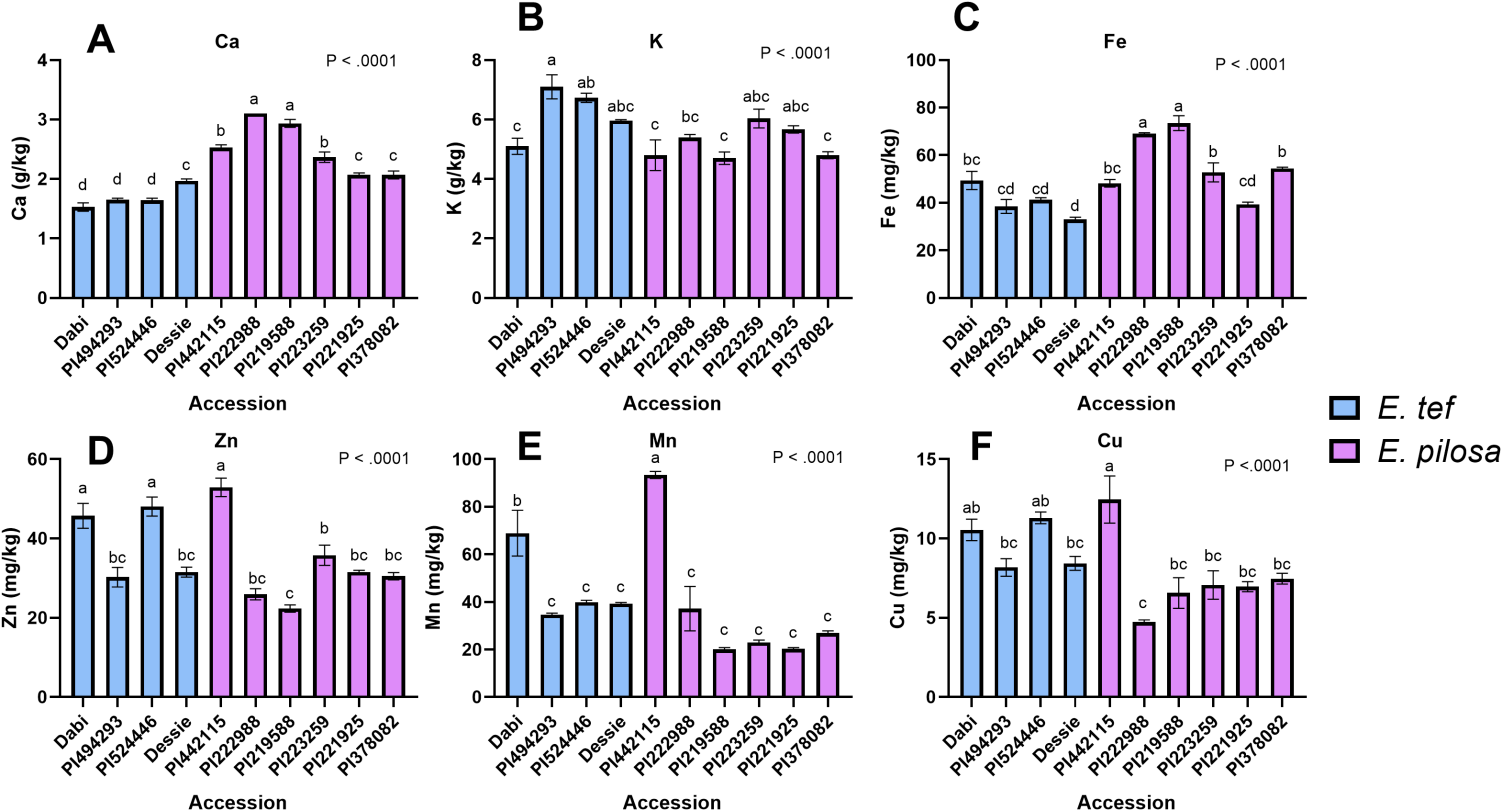
Elemental analysis using ICP-OES. **(A)** Ca, **(B)** K, **(C)** Fe, **(D)** Zn, **(E)** Mn, and **(F)** Cu. Blue bars indicate tef accessions and purple bars indicate *E. pilosa* accessions. Data were analyzed by one-way ANOVA. One-way ANOVA found that differences in mineral concentration for Ca, K, Fe, Cu, Mn, and Zn among the tef and *E. pilosa* accessions were statistically significant (*p< 0.0001).*The SNK *post-hoc* test was used for mean comparison. Bars bearing different letters are significantly different (*p< 0.05*).

To understand the association between seed size and mineral content, we analyzed the mineral concentration of tef and *E. pilosa* accessions contrasting in seed size, along with a few reference varieties of tef using ICP-OES. As shown in **Figure 6**, the differences in all mineral concentrations were statistically significant (*p< 0.0001*). The Ca concentrations in most *E. pilosa* accessions were higher than the tef accessions. The highest Ca concentration (3 g/kg) was detected for PI222988 and PI219588 (**Figure 6A**). The lowest Ca level (1.5 g/kg) was detected in the reference tef cultivar Dabi, along with the tef accessions with small seeds (PI494293), and large seeds (PI524446). The K concentration varied from 6.5-7.5g/kg for all accessions, and the highest concentration was detected for tef accession PI494293 (**Figure 6B**). The Fe concentration of two *E. pilosa* accessions PI219588 (73.47 mg/kg) and PI222988 (69.03 mg/kg) was significantly higher than tef and other *E. pilosa* accessions (**Figure 6C**). Seed Mn concentration was highest for PI442115 (93.37 mg/kg) followed by Dabi (68.91 mg/kg) (**Figure 6E**) while the concentration of Zn and Cu was higher for PI442115, P524446, and Dabi compared to the other accessions (**Figures 6E, F**). Our findings show that accession PI442115 tends to accumulate more micronutrients (Mn, Zn and Cu) than the other *E. pilosa* accession, but only some of the tef accessions. On the other hand, there was no marked difference in P and Mg concentration among the accessions (**Supplementary Figure 1**) while the concentration of S and B was higher in PI442115 (**Supplementary Figure 1**).

Next, using the ICP-OES data discussed above, we analyzed the relationship between seed size and mineral concentration. The seed area was plotted against mineral concentration, and a simple linear regression analysis was conducted to determine if there was a correlation between seed size and mineral content in tef or *E. pilosa*. We found no significant relationship between tef seed area and P (*p = 0.131; R = 0.87*), K (*p = 0.57; R = 0.43*); Ca (*p = 0.84; R = 0.17*), and Mg (*p = 0.64; R = 0.36*) concentration (**Supplementary File 4**; **Supplementary Figure 2**). We detected a significant positive correlation between tef seed area and S concentration (*p< 0.01; R = 0.99*) (**Supplementary Figure 2**). Among the micronutrients, we detected no significant relationship between tef seed area and Fe (*p = 0.59; R = 0.41*), Mn (*p = 0.57; R = 0.43*), Zn (*p = 0.12; R = 0.88*), Cu (*p = 0.10; R = 0.89*), or B (*p = 0.32; R = 0.68*) concentration. We found no significant association between *E. pilosa* seed area and P (*p = 0.25; R = 0.56*), K (*p = 0.71; R = 0.20*), Ca (*p = 0.14; R = 0.67*), and S (*p = 0.052; R = 0.89*) concentration. We detected a significant but weak negative correlation between *E. pilosa* seed area and Mg concentration (*p< 0.05; R = 0.89*). Lastly, we found no significant relationship between *E. pilosa* seed area and Fe (*p = 0.65; R = R = 0.24*), Mn (*p = 0.126; R = 0.69*), Zn (*p = 0.40; R = 0.42*), and Cu (*p = 0.42; R = 0.41*) concentration.

### 3.3 SXRF imaging

Inductively coupled plasma optical emission spectrometry (ICP-OES) is a useful strategy for quantifying minerals in different plant tissues including seeds. However, this method does not provide information on the spatial distribution of mineral elements in those tissues. Therefore, we used synchrotron-based X-ray fluorescence (SXRF) microscopy to visualize minerals in the mature seeds. This method provides semiquantitative elemental distribution at high sensitivity and high spatial resolution (Donner et al., 2012, 2013). Here we report the results of SXRF imaging of the reference tef variety Dabi, small (PI494293) and large (PI524446) seed tef accession, and the *E. pilosa* accession with the smallest seed (PI442115).

**Figure 7:**
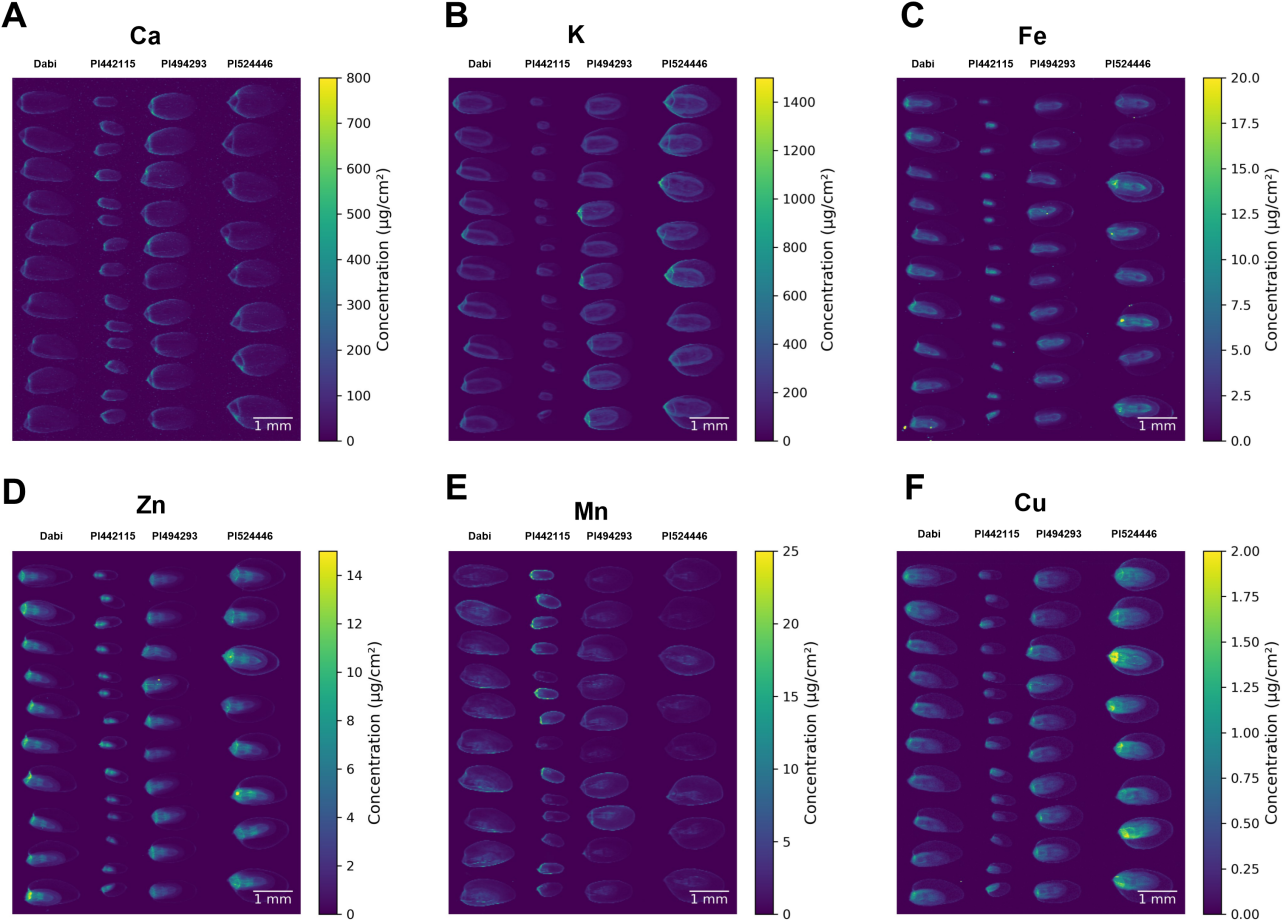
SXRF images of tef and *E. pilosa* seeds. Images show elemental localization of **(A)** Ca, **(B)** K, **(C)** Fe, **(D)** Zn, **(E)** Mn, and **(F)** Cu. Seeds of the same variety are aligned as a column. Accessions left-to-right: (1) Dabi; tef, (2) PI442115; *E. pilosa* (3) PI494293; tef (4) PI524446; tef.

SXRF imaging detected calcium (Ca) apparently localized to the seed coat and was detected in the embryo (**Figure 7A**). It is barely detected in the endosperm. The Ca signal is lower in the reference cultivar Dabi, while there is no marked difference between PI494293, PI524446, and PI442115. When Ca concentration was quantified, the median concentration of Ca was lower in Dabi and slightly elevated in PI442115, with no clear difference between PI494293 and PI524446 (**Supplementary File 5**). Potassium (K) was detected in the outer embryo and the seed coat while it appears absent from the endosperm (**Figure 7B**). For K, we also observed a substantial difference among the accessions. The K signal was stronger in accession PI494293 and PI524446 while it was weakest in the small-seeded *E. pilosa* accession PI442115. Quantification of K concentration showed that PI494493 and PI524446 exhibit elevated median levels of K, in comparison to Dabi and PI442115 (**Supplementary File 5**). Furthermore, there is a greater proportion of signals that exhibit high levels of K in PI494293 and PI524446 in comparison to the other accessions. It should be noted that since the K and Ca SXRF lines have low energy, these signals are measured mainly from the surface of the seed, on the order of 10s of µm. For heavier elements, SXRF signals are measured from greater depths (100s to 1000 µm).

Iron (Fe) is detected in the embryo and the seed coat (**Figure 7C**). There is no marked difference in the signal intensity of Fe among the accessions, although it is not visible in the seed coat of PI442115, which could be due to its small seed size. Quantification of Fe shows that there is no difference in median levels of Fe among the accessions in our sample (**Supplementary File 5**). Like Fe, zinc (Zn) is also localized in the embryo, and the seed coat (**Figure 7D**). The signal of Zn looks slightly higher in PI524446, followed by Dabi, compared to accessions PI494293 and PI442115. Quantification of Zn shows that median levels of Zn are slightly higher in PI524446, but otherwise there is no difference detected in other accessions (**Supplementary File 5**). The manganese (Mn) signal was higher in PI442115, followed by Dabi, where it is detected in the embryo and the seed coat (**Figure 7E**). The Mn signal was lower in the other accessions. Quantification of Mn showed that the median levels of Mn are elevated in Dabi and PI442115, but that a larger proportion of detected signals exhibit high levels of Mn in PI442115 (**Supplementary File 5**). The signal of copper (Cu) was higher in PI524446 as compared to Dabi and accessions PI494293 and PI442115 (**Figure 7F**). Quantification of Cu indicated elevated levels of Cu in PI524446, and a greater proportion of signals from PI524446 show higher levels of Cu than the other accessions (**Supplementary File 5**). Cu is detected in the embryo and the seed coat. Overall, seed size does not appear to affect the general patterns of mineral localization in the seed.

### 3.4 Comparative genomics and functional annotation of seed size regulating genes in rice

This study established that there is natural intraspecific variation in seed size, which indicates that there is likely a genetic basis for this trait. It is known that there are a number of factors that influence final grain size, including numerous genetic factors that are under scrutiny (Li et al., 2022). We hypothesized that the mechanisms regulating seed size in both tef and rice are potentially similar, with other studies indicating high conservation of seed size regulating mechanisms among the cereals (Tao et al., 2020a; Long et al., 2024; Zhang et al., 2015). We gathered the sequences of genes reported to influence grain size in rice and identified putative orthologs in tef using the rice genes as a reference. Although C_4_ grasses such as the millets and sorghum are more closely related to tef, the genetic mechanisms regulating seed size are less established in these species. The mechanisms regulating these traits are better characterized in rice. As a result, we chose to use rice as the reference.

72 seed size regulating genes in rice were collected, representing numerous regulatory mechanisms and gene families, and identified putative orthologs in tef. Then, we conducted a phylogenetic analysis to determine the similarity of the tef sequences to highly similar sequences from other common cereals. The phylogenetic analysis was limited to four gene families, including G-protein pathway genes, mitogen-activated protein kinase (MAPK) signaling, the ubiquitin-proteasome pathway, and brassinosteroid (BR) signaling and biosynthesis, all of which have been shown to influence grain size in rice. Lastly, we conducted a functional analysis of reported seed size regulating genes in rice to establish which mechanisms were most represented and highly influential in rice seed size regulation. For the analysis we used a list of seed size regulating genes from rice, analyzed against the background rice genome. The genes were clustered by the gene ontology (GO) term Biological Processes. Only genes that have been functionally cloned and characterized from rice were included in the list. For those focused on tef breeding, genes of highly enriched pathways may be a key starting place for gene manipulation in tef, if the mechanisms regulating seed size in both cereals are similar.

**Figure 8:**
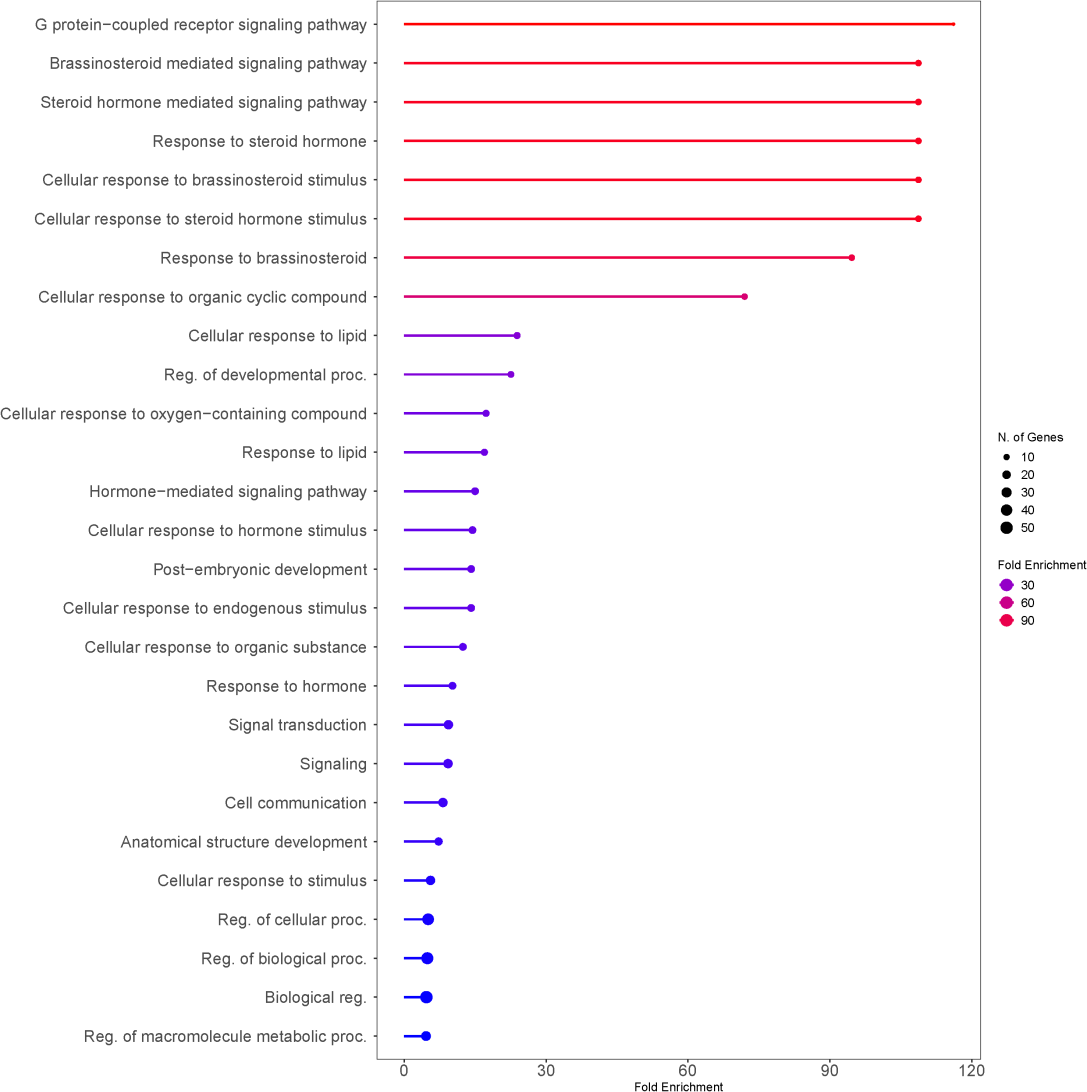
Number of genes that are strongly enriched in various categories of Biological Processes GO term using rice (*Oryza sativa* var. *japonica*) as a model. Reported seed size regulating genes from rice were listed and analyzed against the rice genome background (Oryza sativa Japonica Group genes IRGSP-1.0; Taxonomy ID: 39947). Genes were sorted by GO Biological Processes using ShinyGO v0.80. The size of the circle indicates the number of genes grouped into the biological function. Fold enrichment indicates the genes from our list that are overrepresented in the given pathway as a relationship to the background (Ge et al., 2020).

**Figure 9:**
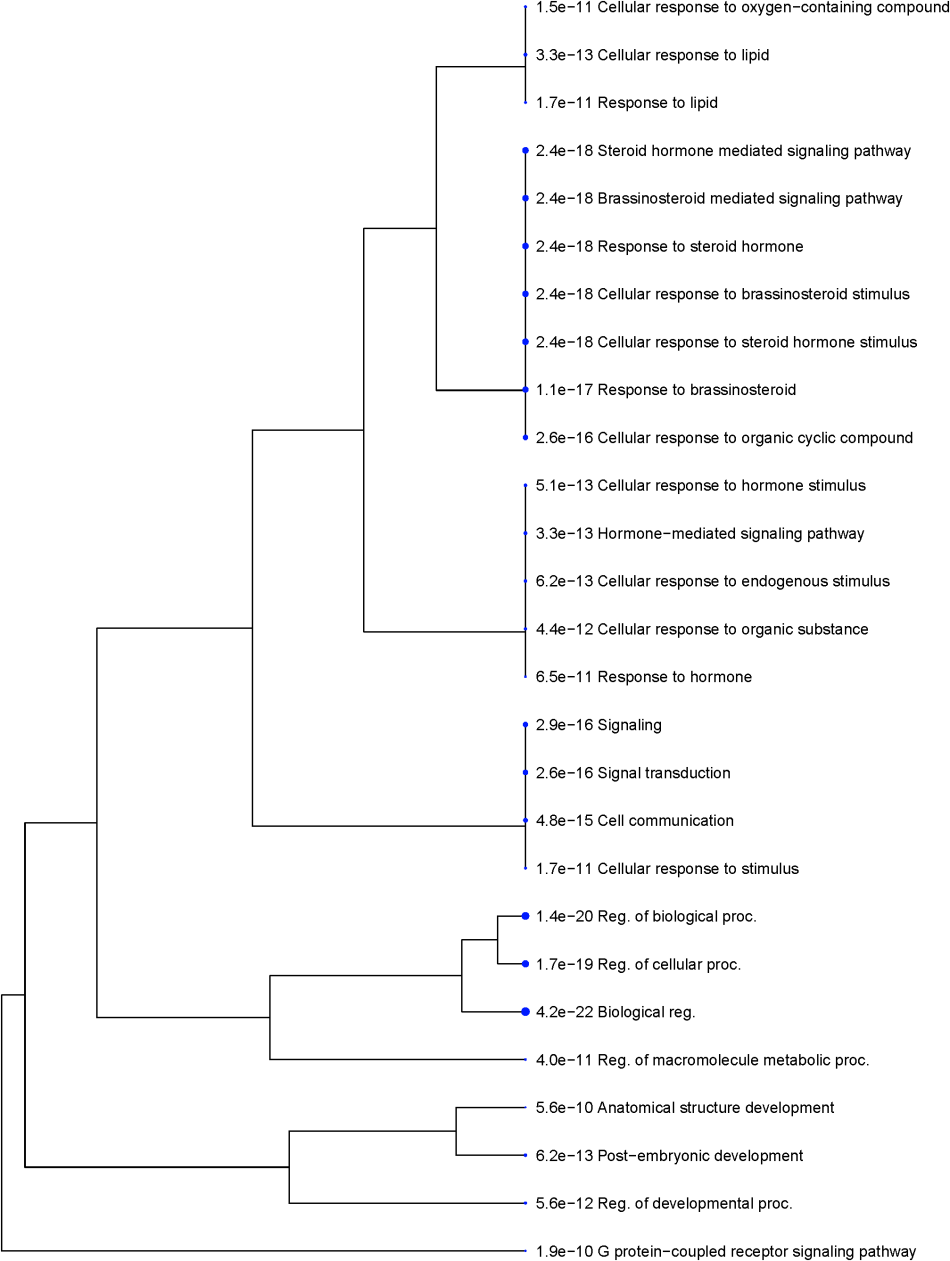
Functional clusters of genes are strongly enriched in distinct categories based on biological function. The size of the circle indicates the number of genes enriched in the group. GO terms of similar biological functions are clustered together. The numerical value in front of the GO ID and term indicates the enrichment False Discovery Rate (FDR; *FDR< 0.05*), which is an adjusted *p - value* to test the statistical significance of the enrichment. Reported seed size regulating genes from rice were listed and analyzed against the rice genome background (Oryza sativa Japonica Group genes IRGSP-1.0; Taxonomy ID: 39947).

The functional analysis of seed size regulating genes suggested that G-protein-coupled receptor signaling (*FDR = 1.9E-10*) was the most significantly enriched biological process, followed by brassinosteroid-mediated signaling (*FDR = 2.4E-18*) (**Figures 8**, **9**). The other highly enriched biological processes involve steroid hormone signaling and response. This indicates that BR signaling, BR biosynthetic regulation, and G-protein mediated signaling are highly important for the regulation of seed size in rice.

**Figure 10:**
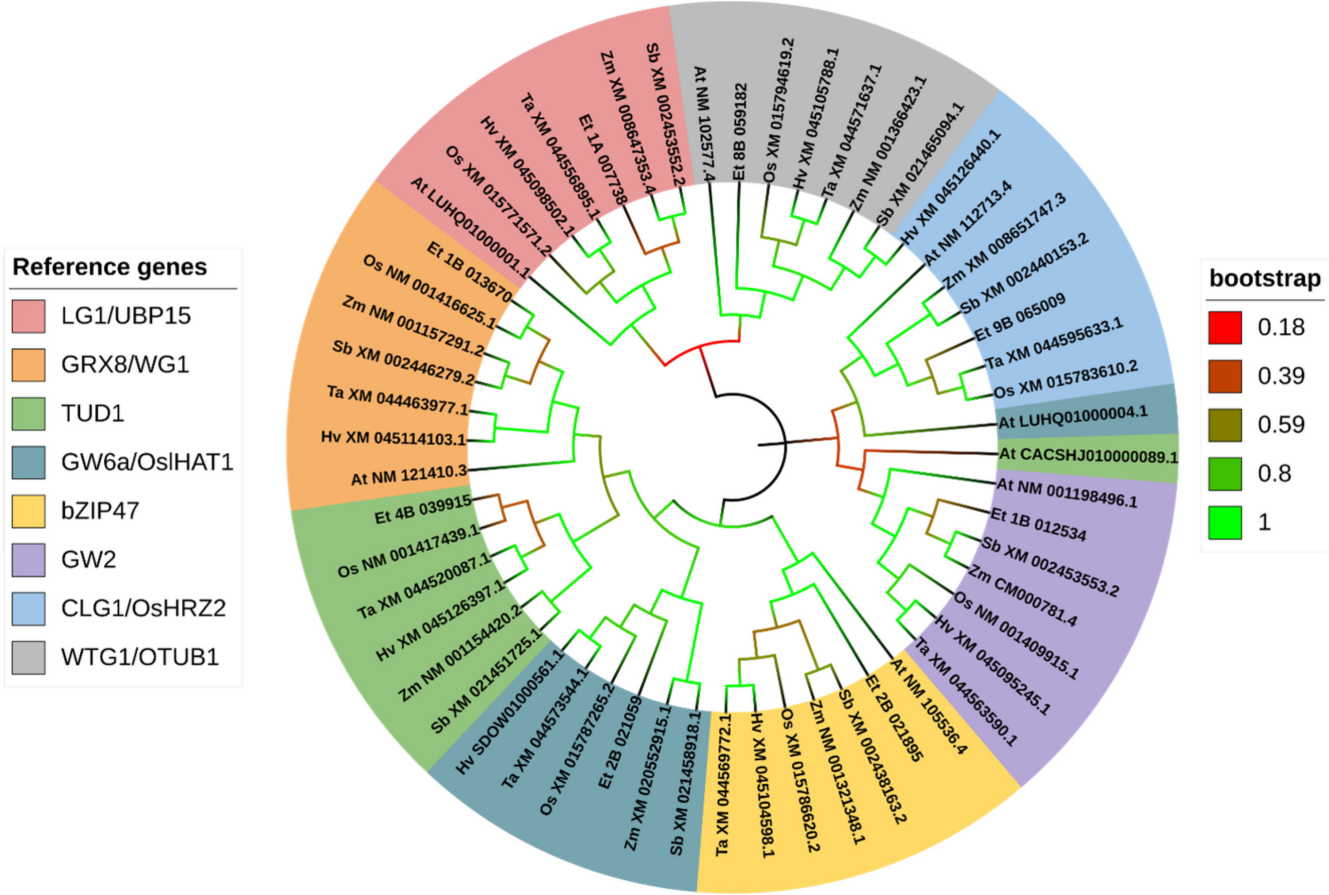
Gene phylogeny of ubiquitin-proteasome pathway genes regulating seed size. Genes were gathered from six monocot species (*Oryza sativa:* Os, *Eragrostis tef*; Et, *Hordeum vulgare*: Hv; *Triticum aestivum*: Ta; *Sorghum bicolor*: Sb; and *Zea mays*: Zm) and the model dicot species (*Arabidopsis thaliana*; At), using the rice sequence as the reference, unless otherwise noted. The first two letters in front of the gene ID represent the initials of the genus and species names. The *Arabidopsis UBP15* homolog LUHQ01000001.1 refers to CDS of the protein OAP15376.1. The *Arabidopsis GW6a/OslHAT* homolog LUHQ01000004.1 refers to CDS of the protein OAO96749.1. Genes are sorted and colored based on the rice gene they were referenced from. In the legend, gene names separated with a forward slash (/) indicate synonymous names. Gene names separated with a plus sign (+) indicate genes that were referenced from more than one rice sequence. Branches on the tree are colored to highlight the bootstrap value.

**Figure 11:**
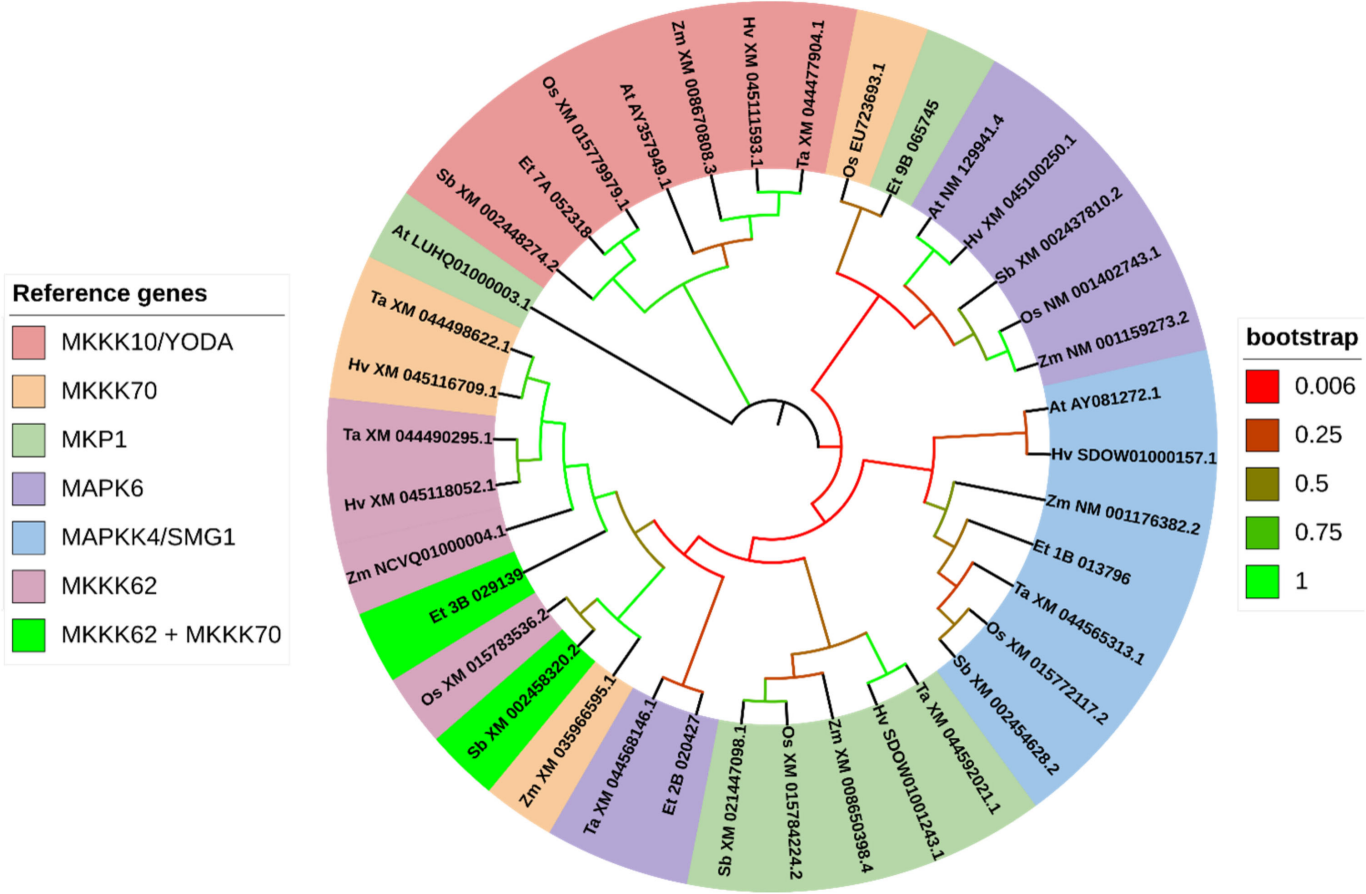
Gene phylogeny of genes associated with MAPK-mediated regulation of seed size. Genes were gathered from six monocot species (*Oryza sativa:* Os, *Eragrostis tef*; Et, *Hordeum vulgare*: Hv; *Triticum aestivum*: Ta; *Sorghum bicolor*: Sb; and *Zea mays*: Zm) and the model dicot species (*Arabidopsis thaliana*; At), using the rice sequence as the reference, unless otherwise noted. The first two letters in front of the gene ID represent the initials of the genus and species names. No significantly similar sequences were identified for *MKKK62* and *MKKK70* in *Arabidopsis thaliana*. *MKKK62* homolog Zm NCVQ01000004.1 represents the CDS associated with the protein PWZ31627.1 of *Z. mays* cultivar inbred line Mo17. The *Arabidopsis* homolog of *MKP1*, LUHQ01000003.1, encodes the protein OAP02192. Genes are sorted and colored based on the rice gene they were referenced from. In the legend, gene names separated with a forward slash (/) indicate synonymous names. Gene names separated with a plus sign (+) indicate genes that were referenced from more than one rice sequence. Branches on the tree are colored to highlight the bootstrap value.

**Figure 12:**
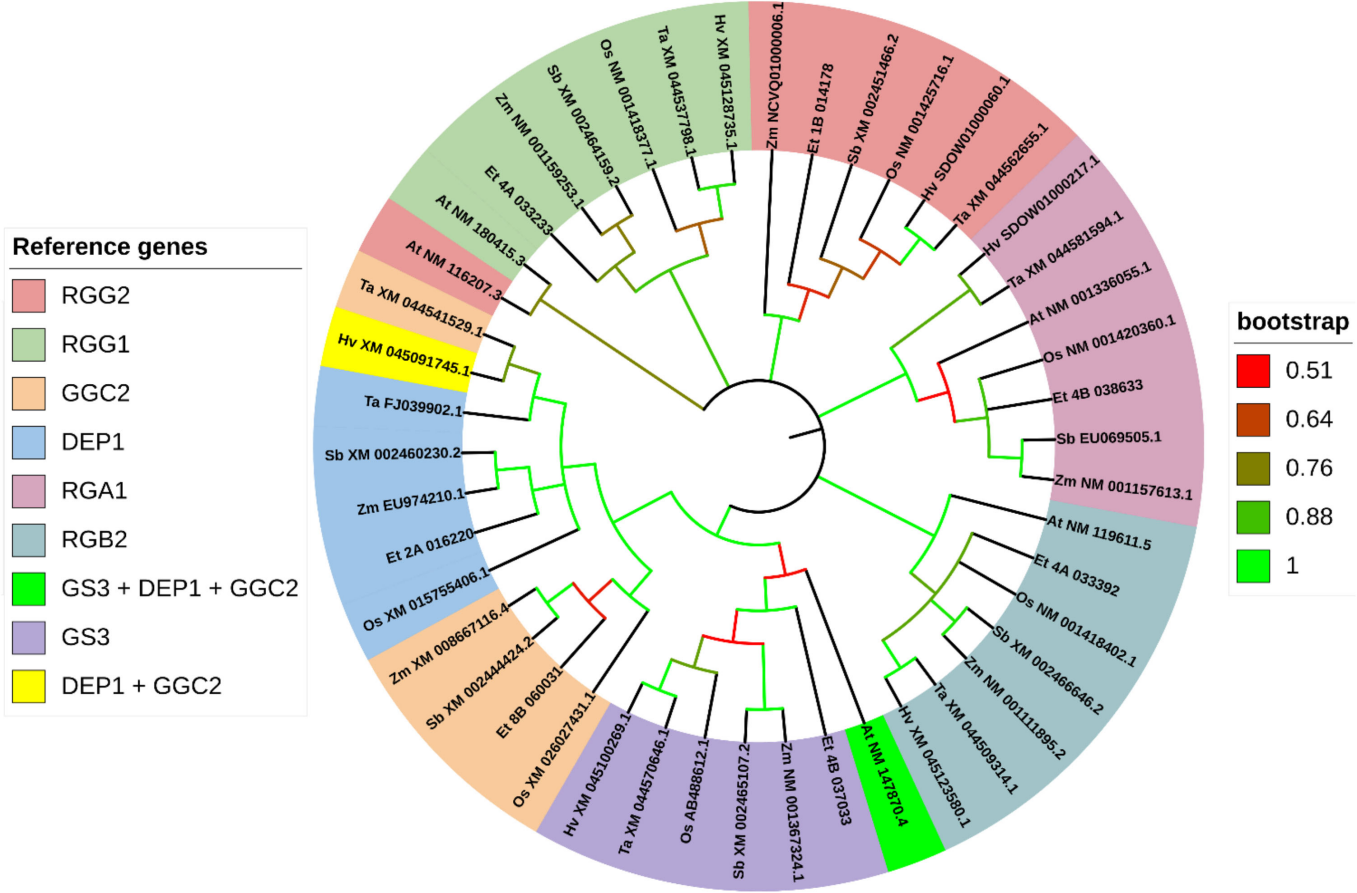
Gene phylogeny of G-protein mediated signaling pathway genes reported to control seed size in rice. Genes were gathered from six monocot species (*Oryza sativa:* Os, *Eragrostis tef*; Et, *Hordeum vulgare*: Hv; *Triticum aestivum*: Ta; *Sorghum bicolor*: Sb; and *Zea mays*: Zm) and the model dicot species (*Arabidopsis thaliana*; At), using the rice sequence as the reference. The first two letters in front of the gene ID represent the initials of the genus and species names. For *RGG1*, no significant ortholog was identified for tef using the rice reference. For *RGG1*, the *Sorghum bicolor* sequence was used to generate the predicted sequence in tef. The *Zea mays* ortholog of *RGG1* generated the same tef sequence. *RGG2* ortholog Zm NCVQ01000006.1 here refers to the CDS associated with the protein PWZ21711.1 of *Z. mays* cultivar inbred line Mo17. Genes are sorted and colored based on the rice gene they were referenced from. In the legend, gene names separated with a forward slash (/) indicate synonymous names. Gene names separated with a plus sign (+) indicate genes that were referenced from more than one rice sequence. Branches on the tree are colored to highlight the bootstrap value.

Based on the phylogenetic analysis, most genes reported to control grain size in rice show high similarity to the loci we identified in the tef genome. For all trees, only the most similar tef sub-genome feature was displayed for each gene. In the phylogenetic analysis of genes grouped into ubiquitin-mediated pathways, we clustered a total of 57 sequences from seven plant species, including rice, wheat, maize, sorghum, barley, tef, and *Arabidopsis*. These genes clustered into eight groups (LG1, GRX8, TUD1, GW6a, bZIP47, GW2, CLG1, and WTG1) (**Figure 10**). For genes grouped into the MAPK pathway for the phylogenetic analysis, we gathered a total of 56 sequences from seven plant species. These genes clustered into seven groups (MKKK10, MKKK70, MKP1, MPK6, MAPKK4, MKKK62, MKK62+MKKK70). For the phylogenetic analysis we display one ortholog identified from tef from each of the rice reference sequences except in the MKKK70 cluster, where BLAST for *MKKK62* and *MKKK70* returned identical sequences in tef and sorghum (**Figure 11**). From the G-protein mediated signaling genes, we gathered 47 sequences among the seven species and clustered them into nine groups (RGG2, RGG1, DEP1, RGA1, RGB2, GS3+DEP1+GGC2, GS3, and DEP1+GGC2). Two additional groups formed from BLAST returning identical sequences from *GS3*, *DEP1*, and *GGC2* in *Arabidopsis*, and BLAST returning identical sequences from *DEP1* and *GGC2* in barley. In the G-protein phylogenetic analysis, we report one tef ortholog in seven of the clusters RGG2, RGG1, DEP1, RGA1, RGB2, and GS3 (**Figure 12**). In BR signaling or biosynthetic-family genes, we collected 154 sequences from seven species, which clustered into 22 groups. An additional group resulted from BLAST of *GSK2* and *GSK3* returning a single *Arabidopsis* sequence. For the purposes of the phylogenetic analysis, we report 21 tef putative orthologs. We identified other putative orthologs from tef which showed significant similarity (*E< 1e-30*) with rice genes from other biological processes, but they are not included in our phylogenetic analysis. We have also included the corresponding homologous sub-genome feature from tef. The complete list of putative orthologous sequences that were identified in tef is available in **Supplementary File 2**.

For simplicity, the phylogenetic analysis of ubiquitin-proteasome family genes includes a single tef sub-genome feature with the highest degree of similarity. From genes involved in the ubiquitin-proteasome pathway, we identified putative orthologous sequences from tef for the genes encoding a ubiquitin-specific protease (UBP15; LG1), a RING ubiquitin E3 ligase (HRZ2), an otubain-like protease (OTUB1; WTG1), a CC-type glutaredoxin protein (GRX8;WIDE-GRAIN 1; WG1), a histone acetyltransferase (OslIHAT1; GW6a), a U-box E3 ubiquitin ligase (TUD1), an E3 ubiquitin ligase (GW2), and a bZIP transcription factor (bZIP47) (**Figure 10**). The tef ortholog of *OsUBP15* forms a single cluster with close similarity to the maize and sorghum *UBP15* orthologs, both of which encode UBP15. Our analysis showed a single cluster of genes referenced from *OsGRX8*, with high confidence of similarity between the rice and tef sequences (94.1% bootstrap value). Rice and tef also share some similarities for *TUD1* but to a lesser extent than UBP15. Our analysis indicates that both *TUD1* and *lHAT1* found in rice are conserved among our sampled Poaceae species, with little similarity to the predicted *Arabidopsis* sequences. Tef sequences share the same level of similarity to *OslHAT1* and the predicted barley and wheat orthologs. Similarly, tef shows equal similarity to the rice *bZIP47* and other grass-family *bZIP47* orthologs. *GW2* referenced genes form a single cluster, with the tef ortholog sharing the closest similarity to the maize and sorghum sequences. *CLG1/HRZ2* from rice is highly similar to the wheat sequence and shares a sub-cluster with tef. Oddly, the predicted *CLG1/HRZ1* ortholog in barley is most similar to the sorghum *OTUB1* sequence. The tef ortholog of *OTUB1* shares a high bootstrap value with *OsOTUB1* and other grass-family *OTUB1* predicted orthologs. Our analysis suggests the tef genome contains two copies of each of the aforementioned genes, i.e. two homologous sub-genome features with high degrees of similarity. In tef, we identified an additional, highly similar copy of *GW6a* on chromosome 1A, which was the second closest genomic feature returned from the BLAST results.

We report five orthologs in tef that resemble genes involved in MAPK-mediated regulation of seed size in rice. Tef has at least two homologous sub-genome features for each MAPK-family gene we analyzed. Notably, we found an additional copy of *MKK4* with significant similarity on chromosomes 1A and 1B. The putative tef ortholog of *OsMAPK6* shows low similarity to the rice gene, but closer similarity to the *T. aestivum* (wheat) *MAPK6* ortholog (XM_044568146.1), predicted to encode a MAP Kinase (**Figure 11**; **Supplementary File 2**). Both the tef and wheat orthologs of *MAPK6* form a cluster that shows higher similarity to the clusters for *MAPKKK70* and *MAPKKK62.* The tef ortholog of *MKK4* forms a cluster of MKK’s in wheat, sorghum, rice, corn, and *Arabidopsis*. Our phylogenetic analysis indicates a high confidence of similarity (100% bootstrap value) between the predicted tef *MKKK10* sequence and the rice *MKKK10* sequence and form an obvious cluster with a high bootstrap value with the other five species we analyzed. Interestingly, the predicted tef ortholog of *MKP1* shows close similarity with the rice sequence for *MKKK70*, but next to the cluster of rice *MAPK6* and four other *MAPK6* orthologs. *OsMKKK62* shows high similarity to the sorghum and maize predicted orthologs of *MKKK70*, but little resemblance to the redundant kinase *OsMKKK70*. Both rice sequences *MKKK62* and *MKKK70* returned identical sequences in tef and sorghum. This indicates that tef and sorghum potentially lack the redundant version of *MKKK70*.

Using the sequences for the standard seed size regulating G-proteins in rice (**Figure 12**), we were able to identify all but one of tef putative orthologs using rice sequences. Each G-protein family gene we analyzed returned two homologous sub-genome features in tef. For *RGG1*, we used the sequence from *S. bicolor* (sorghum) (XM_002464159.2) to generate the CDS in tef. From the phylogenetic analysis, the *RGG1* tef ortholog shows no significant similarity to the rice *RGG1*, but high similarity to the *Z. mays* (maize) and sorghum sequences, which are annotated as Gγ proteins. We also predict coding sequences in tef that show high similarity to the rice genes *RGA1* and *RGB2*. In addition to *RGG1*, *DEP1* and *GGC2* orthologous tef sequences show closer similarity to the maize and sorghum sequences, than to the rice sequences. The tef ortholog of *GS3* shows lower similarity to the rice sequence but shares a high bootstrap value with a cluster that includes the *GS3* gene in rice, in addition to the barley, wheat, sorghum, and maize *GS3* putative orthologs.

**Figure 13:**
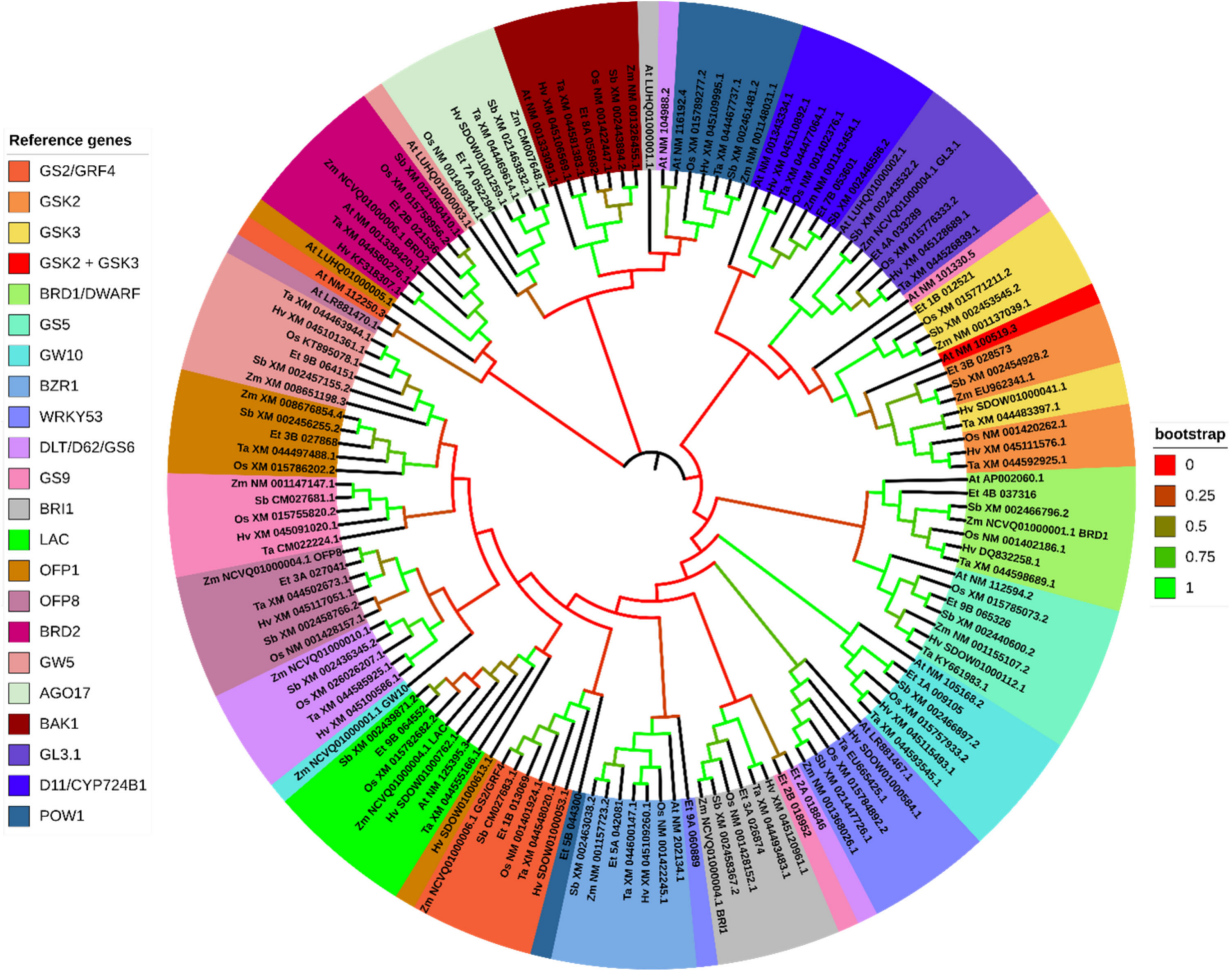
Gene phylogeny of genes involved in brassinosteroid (BR) signaling and biosynthesis reported to control grain size in rice. Genes were gathered from six monocot species (*Oryza sativa:* Os, *Eragrostis tef*; Et, *Hordeum vulgare*: Hv; *Triticum aestivum*: Ta; *Sorghum bicolor*: Sb; and *Zea mays*: Zm) and the model dicot species (*Arabidopsis thaliana*; At). The first two letters in front ot the gene ID represent the initials of the genus and species names. No significantly similar sequences were identified in *Arabidopsis* for the *AGO17* cluster. Some of the sequences in the tree above share a common label (gene ID). Here is the legend for those with common gene IDs: (Zm NCVQ01000006.1 (BRD1): PWZ55818.1) (Zm NCVQ01000004.1 (GL3.1): PWZ31897.1) (Zm NCVQ01000004.1 (BRI1): PWZ33145.1) (Zm NCVQ01000004.1 (LAC): PWZ31747.1) (Zm NCVQ01000004.1 (OFP8): PWZ31834.1) (Zm NCVQ01000006.1 (BRD2): PWZ21402.1) (Zm NCVQ01000006.1 (BRD2): PWZ22989.1) (*BRI1/DG1*: At LUHQ01000001.1: OAP19821.1) (*GL3.1*: At LUHQ01000002.1: OAP09351.1) (*GW5*: LUHQ01000003.1: OAP01951.1) (*OFP1*: LUHQ01000005.1: OAO94238.1). Genes are sorted and colored based on the rice gene they were referenced from. Genes names separated with a forward slash (/) indicate synonymous names. Gene names separated with a plus sign (+) indicate genes that were referenced from more than one rice sequence. Branches on the tree are colored to highlight the bootstrap value.

The phylogenetic analysis of BR pathway associated genes were limited to 21 reference genes (**Figure 13**; **Supplementary File 2**). Fourteen of the reference genes returned two highly similar homologous sub-genome values in tef. We found a significantly similar third copy of *BRD2* on chromosome 6A. *GW5* and *GS5* returned two sub-genome values, but in both cases, other loci on different chromosomes were ahead of the second homolog in similarity. We also found several highly similar copies of *D11*/CYP724B1 along chromosomes 7A and 7B. *GW10* returned a third sub-genome feature on chromosome 5B (*E-value = ~ 0.0*). From the gene phylogeny, we identified a locus in tef that shows high similarity to the rice *GSK2* sequence, along with the sorghum and maize *GSK2* orthologs. The predicted tef *GSK3* ortholog shows high similarity to the maize and sorghum *GSK3* sequences, which both encode SHAGGY-related kinases. *BRD1*, *BRD2, GL3.1, GS5, BAK1* and *BZR1* show a high degree of conservation among the seven species we analyzed, with clear clustering of sequences with high bootstrap values. The cluster of genes generated from the *OsD11*/CYP724B1 sequence shows high similarity among the monocots, with *Arabidopsis* forming an outgroup of the cluster. The tef orthologous sequence of *OsD11*/CYP724B1 was most closely related to the maize gene, which also encodes a CYP724B1. The cluster forming *LAC*-related sequences formed a single cluster with a high bootstrap value. However, within the *LAC* cluster, the separation between the homologs is less clear. Regardless, the tef and sorghum *OsLAC* orthologs show the closest similarity. The *BRI, GW5, GS2/GRF4, OFP1, AGO17* and *OFP8* clusters indicate high conservation among the monocots, with low similarity to the *Arabidopsis* sequences. Interestingly, the tef orthologous sequences to *WRKY53* and *POW1* show low similarity to the rice and other sequences we analyzed. The tef ortholog of *GS9* sequences appeared to be a partial sequence and shows low similarity to the main cluster with *OsGS9* and *GS9* orthologs in the other monocots. The tef *GS9* and *DLT* sequences form a two-leafed group in our analysis, both of which are not included in the main clusters. The cluster for *GW10* forms a clear group, including *Arabidopsis*, with a 99.2% bootstrap value. Oddly, the maize *GW10* ortholog is much more distant. The sorghum *GW10* sequence is predicted to encode CYP450-89A2, the same as *OsGW10* and exhibits the highest similarity to the putative tef ortholog.

## 4 Discussion

The purpose of this research was to explore the natural variation in seed size among 189 unique genotypes of tef. From our analysis, we have found significant variation in seed length, width, and area among most of the tef population. Using representative accessions, we also found significant variation in 1000-grain weight. Our research also led us to explore the seed size variation in the wild progenitor of tef, *E. pilosa*. Additionally, tef’s nutritional qualities confer special attention, due to its superiority over more popular cereals such as rice, wheat, and maize (Ligaba-Osena et al., 2021). As such, we studied the overall mineral content in seeds of selected tef and *E. pilosa* grains and explored their distribution within the seeds using SXRF imaging. Lastly, we performed comparative genomics to identify putative seed size regulating genes in tef, conducted a phylogenetic analysis of seed size regulating genes across a variety of plant species, and conducted functional annotation of seed size regulating genes based on genes from the model monocot cereal *Oryza sativa* (rice).

### 4.1 Mineral concentration and spatial distribution in the grains

Studying the spatial distribution of minerals in seeds is important for understanding the fundamentals of seed development, nutrient bioavailability, bio-accessibility, and strategies for biofortification. Cereals provide a substantial portion of the mineral nutrients acquired through diet (Arafsha et al., 2023) and mineral malnutrition remains a global health concern and is widespread in both the developed and developing world (Zimmermann and Hurrell, 2007). To study the mineral content of biological samples such as grains, inductively coupled plasma mass spectrometry (ICP-MS) or ICP-OES has been widely used (Dame, 2020; Woldetsadik et al., 2024). However, it gives no insight into localization patterns and potential bioavailability.

To study the spatial distribution of minerals, researchers have turned to synchrotron-based x-ray fluorescence (SXRF) imaging to visually study and quantify the minerals within biological tissues. XRF imaging is a non-destructive imaging technique that allows *in situ* 2D quantification of elements (Donner et al., 2012, 2013). Some studies have utilized XRF spectroscopy to study hyperaccumulators (Goudard et al., 2024). Others have utilized XRF imaging for studying micronutrient mobilization during seed germination *in vivo* (Takahashi et al., 2009). SXRF microscopy has proven to be useful in studying alterations in mineral accumulation in loss of function studies (Chia et al., 2023; Sheng et al., 2021). In this report, we utilized SXRF imaging to explore the distribution of minerals within the tef grains for the first time. Our findings show that Ca is associated with the seed coat and the embryo while K is detected in the outer embryo and seed coat, and the micronutrients are predominantly associated with the embryo and seed coat. The concentration of minerals was also variable among the genotypes we analyzed (**Figure 5**). A similar distribution pattern has been reported in rice, where minerals were shown to localize in the embryo and remain absent from the endosperm (Lu et al., 2013).

For processing and consumption, mineral localization is nutritionally important. In many cereals, the milling process separates the embryo and endosperm (Shewry et al., 2020). Because mineral localization is variable, this may inadvertently remove micronutrients from the final product. The whole seed is milled in some cereals, such as tef, leaving the endosperm and embryo components together. Milling the entire seed potentially increases the overall amount of nutrients in the flour. However, the localization of minerals in different seed tissues may alter bio-accessibility or dictate how easily minerals are accessed from seed tissues upon digestion (Arafsha et al., 2023). For example, the plant cell wall is resistant to digestion in the human digestive tract. The cells of the aleurone layer are composed of water-insoluble indigestible fibers (Arafsha et al., 2023; Brouns et al., 2012). The starchy endosperm is the most digestible part of the seed, but lacks minerals, which was evident in the tef accessions we analyzed (Gidley, 2024; Khalid et al., 2023) (**Figure 5**). Furthermore, the accessibility of minerals can be affected by how the mineral is stored in the seed chemically. For example, prominent levels of phytate can bind minerals in the seeds, making them less accessible during digestion (Mandha and Raes, 2023). Phytate tends to localize at the highest concentration in the aleurone layer and the embryo (Bohn et al., 2008). Baye et al. (2015) addressed this issue though the additions of phytate degrading enzymes in tef flour to improve iron bioavailability. Furthermore, tef is usually consumed after fermentation, which was reported to decrease the concentration of antinutrients such as phytate and increase mineral bioavailability (Fischer et al., 2014; Shumoy and Raes, 2017).

### 4.2 Evolution of seed size and mineral content of tef

Plant domestication has followed convergent, but highly similar processes globally among many cultivated crops (Fuller et al., 2014). In cereals, the major traits selected were shattering and lodging resistance, along with greater seed size to improve yield and quality. Many domestication traits were selected for purposely, whereas others may have been inadvertently acquired through genetic linkage, pleiotropy, or otherwise unconsciously (Purugganan, 2019). The increase in seed size among the cereals is contested, with some arguing that enlarged seed size was consciously selected for, which would bring higher yield and ease of handling. Others have argued that seed size was linked to characteristics such as height, suggesting that the enlargement of seed size was unconscious (Purugganan, 2019; Meyer and Purugganan, 2013). In our study, we show that tef seeds can be as much as 4.44 times larger by area and 2.35 times longer than its progenitor *E. pilosa*. The evolution of grain size in tef remains to be fully understood.

In some cases, individual genes that produce highly desirable traits and share commonality among closely related cultivars or between species, show signs of selection at the genetic level (Meyer and Purugganan, 2013). The capability to sequence genomes reliably and efficiently for genome wide association studies (GWAS) and quantitative trait loci (QTL) mapping has provided novel insights to the genomic evolution of domesticated cereals. In rice, this has revealed the genetic basis of seed size, a key determinant of yield in cereals (Tao et al., 2020b). We anticipate that future research will reveal such insights in tef.

Seed size is an extraordinarily complex trait that is influenced by both genetic and environmental conditions. Furthermore, there seems to be a tradeoff between offspring number and size, as reported by those studying natural variations and single gene mutants (Gnan et al., 2014; Zhang et al., 2017a, 2017b; Xiao et al., 2019). In natural populations, there is strong environmental pressure that determines seed size, and variation exists across species (Venable, 1992). Razzaque et al. (2023) showed that local adaptation to climate was a major predictor of seed size of the perennial grass *Panicum hallii*. They showed that annual temperature showcased the strongest predictability in determining seed size among *P. hallii* ecotypes. During the course of domestication, humans artificially selected against the natural plant dispersal mechanisms (shattering) and selected for increased seed size (Harlan et al., 1973). Harlan et al. (1973) revealed that the transition from wild grasses to their domesticated counterparts follow highly similar trajectories among species. Fuller et al. (2014) substantiated these ideas, highlighting extensive parallelism (convergence) in the evolution of non- shattering species and the enlargement of seeds in cereals. In tef, one estimate indicates a 72% difference in grain thickness in tef and *E. pilosa*, which is consistent with our estimates (D’Andrea, 2008). D’Andrea (2008) speculated that early tef cultivators encountered several obstacles during domestication and that tef’s domestication history was highly unconventional in comparison to other cereals. In speculation about why tef grains have remained so small, one hypothesis suggests that natural introgression with *E. pilosa* may have helped maintain small seed sizes. Early cultivators more likely recognized tef’s susceptibility to shattering and lodging and may have selected against large panicles and large, heavy grains (D’Andrea, 2008; Ketema, 1997). Lastly, water-conservation practices and attempts to reduce lodging resulted in minimal tilling practices in Ethiopia’s semi-arid highlands, which is hypothesized to reduce selection pressure for large seeds (Harlan et al., 1973). Furthermore, D’Andrea (2008) notes that modern Ethiopian farmers do not select for large seeds, but rather for color, appearance, increased panicle branching, and more numerous grains, suggesting that any improvement to grain size in tef has been non-deliberate or unconscious (Purugganan, 2019; Meyer and Purugganan, 2013). For those interested in breeding large seed cultivars of tef, these limitations remain a major point of consideration.

The relationship between seed size and mineral content remains elusive in tef. Previously, there has been some speculation regarding the relationship between seed size and mineral content. It is suggested that higher mineral contents of the seed may confer a major evolutionary advantage. Additionally, smaller seeds are more easily dispersed and evolutionarily beneficial but have become agronomically undesirable in many seed-bearing crops. However, some have suggested that any benefit conferred from a larger seed diminishes if the external nutrient availability is low, when smaller, nutrient-dense seeds may be much more advantageous (Grubb and Burslem, 1998). Curiously, Grubb and Burslem (1998) found an inverse relationship between mineral content and seed size. They showed that larger seed size diminished N, P, K, Mg, and Ca concentrations in several different crop species. It is interesting to speculate that the ancestral species of tef, *E. pilosa*, may have benefited evolutionarily from this kind of selection. However, this remains to be validated. The accession with the smallest and lightest grains from either the tef or *E. pilosa* accessions was PI442115 (*E. pilosa*), which showed the highest concentration of Cu, Zn, and Mn from the ICP-OES, and had clearly higher levels of Mn in the SXRF (**Figures 6**, **7**). However, in PI442115, the mineral concentration of other elements was relatively lower, potentially indicating some kind of trade off. The preliminary results of whether there is any relationship between seed size and mineral concentration in tef or *E. pilosa* seeds are inconclusive. We will continue to pursue this by analyzing the mineral content of more tef genotypes, where we can begin to unravel the relationship between seed size and mineral content further.

### 4.3 Overview of the genetic mechanisms regulating seed size

The genetic mechanisms regulating seed development and seed size are complex and poorly understood. Even in a model monocot such as rice, the underlying mechanisms regulating seed size remain fragmented. Regardless, developing novel rice cultivars with enhanced seed size is a major area of research, as larger seed size is often associated with improved yield and quality. In tef cultivation, its small seed size presents numerous challenges to its cultivation. Much of the general biology, let alone the genetic mechanisms regulating seed development, remains to be elucidated in tef. Additionally, the tef genome remains to be fully annotated.

In rice there are ~80 genes that have been implicated in seed size regulation (Li et al., 2022). Some of these have been selected for over the course of domestication, whereas many others represent rare alleles identified from mutagenic studies or evaluation of rice progenitor species. Most of these genes regulate seed size via changes to cell proliferation, elongation, and enlargement in the seed hull (and related tissues), or in maternally derived tissues such as the embryo or endosperm (Li et al., 2022). Many of the genes encode receptors, proteins involved in various signaling mechanisms, hormone biosynthetic enzymes, transcriptional regulators, and transcription factors. For simplicity, they can be grouped by a shared pathway or function, but many have been shown to be multi-pathway regulating or multifunctional.

Our analysis suggests that G-protein mediated signaling is the most significantly (*FDR< 0.05*) enriched biological process regulating grain size in rice (**Figures 8**, **9**). Several G-proteins have been implicated in seed size regulation in rice, many of which were identified as major quantitative loci. The rice genome encodes one Gα subunit (RGA1), one Gβ subunit (RGB1), and five Gγ subunits (RGG1, RGG2, GS3, DEP1, and GGC2), which compose the major G-proteins in rice (Mao et al., 2010; Fan et al., 2006; Sun et al., 2018; Tao et al., 2020b; Miao et al., 2019). Plants also exhibit a number of non-canonical extra-large GTP-binding proteins (XLGs), which are large atypical Gα subunits (Maruta et al., 2021). XLGs have been shown to regulate several complex traits including panicle architecture, stress tolerance, and some agronomic traits such as 1000-grain weight (Biswal et al., 2022; Zhao et al., 2022; Cantos et al., 2023). However, compared to the other G-proteins we used in our analysis, these XLGs are not as well characterized and their effects on seed size regulation are still under active investigation. As a result, we chose to focus our study on the better characterized G-protein subunits and omit XLGs from our analysis. Unlike animals, plants lack G-protein coupled receptors (GPCR’s) and are self-activated (Urano and Jones, 2014). To transduce the signal, Gβ and Gγ subunits form a Gβγ heterodimer, which go on to interact with Gα (Maruta et al., 2021; Urano and Jones, 2014). Evidence suggests that various combinations of G-protein subunits exhibit unique alterations to key developmental processes and specific alterations to various grain size attributes, either positively or negatively (Sun et al., 2018; Pandey, 2020). We were able to identify putative orthologs in tef for all but one of the G-protein family genes using the rice CDS (Criterion from BLAST: *E< 1e-30*). We found no significantly similar sequences in tef using the *OsRGG1* sequence but found that the maize and sorghum *RGG1* orthologs produced the same tef sequence, which was validated by our phylogenetic analysis (**Figure 12**). Otherwise, the other tef sequences we identified pass the standard cutoff for the determination of homology using BLAST (Choudhuri, 2014; Pearson, 2013).

The ubiquitin-proteasome pathway regulates protein stability, activity, and degradation in eukaryotes. In plants, ubiquitin-mediated processes contribute to many biological processes, such as embryogenesis, hormone signaling, and senescence (Moon et al., 2004). Eight genes involved in ubiquitin-mediated regulation of seed size have been reported in rice. Many of these genes have been identified in other grasses and in *Arabidopsis*, where they have exhibited highly conserved functions (Du et al., 2014; Tang et al., 2023; Shi et al., 2019; Keren et al., 2020; Li and Li, 2014; Wang et al., 2020; Zombori et al., 2020; Brinton et al., 2018). Most of the genes grouped in ubiquitin-mediated pathways act as regulators of other systems, and exhibit crosstalk with the G-protein, BR, GA, and MAPK pathways, in addition to regulating the activity of some transcription factors (Bai et al., 2024; Yang et al., 2021; Hu et al., 2013; Hao et al., 2021; Tian et al., 2021). From tef, we report eight putative orthologs involved in ubiquitin-mediated regulation of grain size, along with their homologous sub-genome feature (**Figure 10**; **Supplementary File 2**) (Choudhuri, 2014; Pearson, 2013).

The mitogen activated protein kinase (MAPK) cascade is a central signaling mechanism in eukaryotes (Taj et al., 2010). MAPK cascades are comprised of closely related kinases that are sequentially phosphorylated, eventually leading to a final activated MAPK that phosphorylates transcription factors and other transcriptional regulators (Hardie, 1999). In rice, a three-gene MAPK module serves as a positive regulator of grain size (Liu et al., 2021; Xu et al., 2018; Liu et al., 2015; Duan et al., 2014), with a single phosphatase acting as the negative regulator of the module (Guo et al., 2018; Xu et al., 2018). Furthermore, two redundant MKKK’s have been shown to regulate the activity of the transcription factor *OsWRKY53* and influence grain size via the MAPK module mentioned above (Liu et al., 2021; Tian et al., 2021). Of the six MAPK-related seed size regulating genes reported in rice, only five putative orthologs could be described in tef, along with their sub-genome homolog (**Figure 11**; **Supplementary File 2**). BLAST of the tef genome with *OsMKKK62* and *OsMKKK70* returned the same tef sequence, indicating that the tef genome likely lacks the paralog of this kinase.

Several phytohormones and their associated regulatory mechanisms have been linked to seed development, including brassinosteroids (BRs), auxin (IAA), gibberellins (GA), and cytokinin’s (Li et al., 2022). The functional analysis concluded that brassinosteroid signaling and homeostasis was a significantly enriched biological process involved in seed size regulation in rice (**Figures 8**, **9**; **Supplementary File 3**). We report 21 putative orthologs in tef from rice genes involved in BR biosynthesis and regulation, along with their sub-genome homolog (**Figure 13**). Furthermore, several transcription factors, functional proteins, microRNAs (miRNA’s), and proteins involved in endosperm development are reported to determine final grain size and weight in rice. Of such genes, we report 29 putative orthologs in tef, along with their corresponding sub-genome homolog (**Supplementary File 2**).

### 4.4 Conclusions and limitations

This study characterized the seed size phenotype of 189 tef and 11 *E. pilosa* genotypes. Unfortunately, this is reflective of most, but not all of the tef population available at the USDA-ARS germplasm center. We feel our analysis likely reflects the true nature of the tef population but remains incomplete without more research. In our analysis we only utilized tef and *E. pilosa* accessions whose seeds exhibited relatively homogenous coloration and treated samples with different colored seeds as impure. To ensure that our seed size analysis was as accurate as possible, we excluded samples with multi-colored seeds. Additionally, the seeds used in this study were from stock collections from the U.S. National Plant Germplasm Center to ensure they were sourced from a single location, grown under the same field conditions. Notably, replication of many of these accessions under greenhouse conditions indicated that seed size is true to type (data not shown).

Furthermore, there is no literature on the anatomy of tef seeds, so the conclusions we make for the SXRF imaging are highly general. To better understand seed development in tef, the impact of genetic manipulation, and the localization of minerals, more research into the developmental anatomy of tef is required. Moreover, the tef genome annotation remains to be completed, so validation of the findings from our comparative genomic analysis is impossible without further research. Future research into tef should aim to address these limitations. Regardless, we hope the findings from this research can provide a starting point for those interested in breeding high quality, nutritious tef, and for those interested in the evolutionary genetics of tef domestication.

## Data Availability

The datasets presented in this study can be found in online repositories. The names of the repository/repositories and accession number(s) can be found in the article/**Supplementary Material**.
