# Supplementary material for: Biggest of tinies: natural variation in seed size and mineral distribution in the ancient crop tef [Eragrostis tef (Zucc.) Trotter]
Source: Front Plant Sci. 2024 Dec 12;15:1485819. doi: 10.3389/fpls.2024.1485819 (PMC11669528; doi:10.3389/fpls.2024.1485819)
Supplement: Supplementary file 1 [file DataSheet1.zip › supplemental-files/newick-files/phylogentic_analysis_seed_size_genes.html]

Gene trees for seed size regulating genes


# Gene trees for seed size regulating genes

Author

Eric Whisnant

```
library(ape)
library(ggtree)
```

```
ggtree v3.12.0 For help: https://yulab-smu.top/treedata-book/

If you use the ggtree package suite in published research, please cite
the appropriate paper(s):

Guangchuang Yu, David Smith, Huachen Zhu, Yi Guan, Tommy Tsan-Yuk Lam.
ggtree: an R package for visualization and annotation of phylogenetic
trees with their covariates and other associated data. Methods in
Ecology and Evolution. 2017, 8(1):28-36. doi:10.1111/2041-210X.12628

Guangchuang Yu. Using ggtree to visualize data on tree-like structures.
Current Protocols in Bioinformatics. 2020, 69:e96. doi:10.1002/cpbi.96

Guangchuang Yu, Tommy Tsan-Yuk Lam, Huachen Zhu, Yi Guan. Two methods
for mapping and visualizing associated data on phylogeny using ggtree.
Molecular Biology and Evolution. 2018, 35(12):3041-3043.
doi:10.1093/molbev/msy194
```

```
Attaching package: 'ggtree'
```

```
The following object is masked from 'package:ape':

    rotate
```

```
mapk_tree <- read.tree("")
```

```
Warning in read.tree(""): empty character string.
```
