# Supplementary material for: Biggest of tinies: natural variation in seed size and mineral distribution in the ancient crop tef [Eragrostis tef (Zucc.) Trotter]
Source: Front Plant Sci. 2024 Dec 12;15:1485819. doi: 10.3389/fpls.2024.1485819 (PMC11669528; doi:10.3389/fpls.2024.1485819)
Supplement: Supplementary file 1 [file DataSheet1.zip › supplemental-files/sup-fig-01.DOCX]

**Supplemental Figure 1**. Additional results from ICP-OES of tef and *E. pilosa* seeds
