# Supplementary material for: Biggest of tinies: natural variation in seed size and mineral distribution in the ancient crop tef [Eragrostis tef (Zucc.) Trotter]
Source: Front Plant Sci. 2024 Dec 12;15:1485819. doi: 10.3389/fpls.2024.1485819 (PMC11669528; doi:10.3389/fpls.2024.1485819)
Supplement: Supplementary file 1 [file DataSheet1.zip › supplemental-files/sup-fig-02.DOCX]

**Supplemental Figure 2.** Plots comparing seed area to mineral concentration for both tef (A and B) and *E. pilosa* (C and D). Rows compare macro- (A and C) and micronutrient (B and D) concentrations from the ICP-OES plotted against either tef (n = 4) or *E. pilosa* (n = 6) seed area measurements. Elements found to have significant correlation are labeled with a star (*, p < 0.05; **, p < 0.01). The analysis summary can be found in Supplemental File 4.
