# Supplementary material for: Biggest of tinies: natural variation in seed size and mineral distribution in the ancient crop tef [Eragrostis tef (Zucc.) Trotter]
Source: Front Plant Sci. 2024 Dec 12;15:1485819. doi: 10.3389/fpls.2024.1485819 (PMC11669528; doi:10.3389/fpls.2024.1485819)
Supplement: Supplementary file 1 [file DataSheet1.zip › supplemental-files/supplemental-File-05.PPTX]

## Slide 1
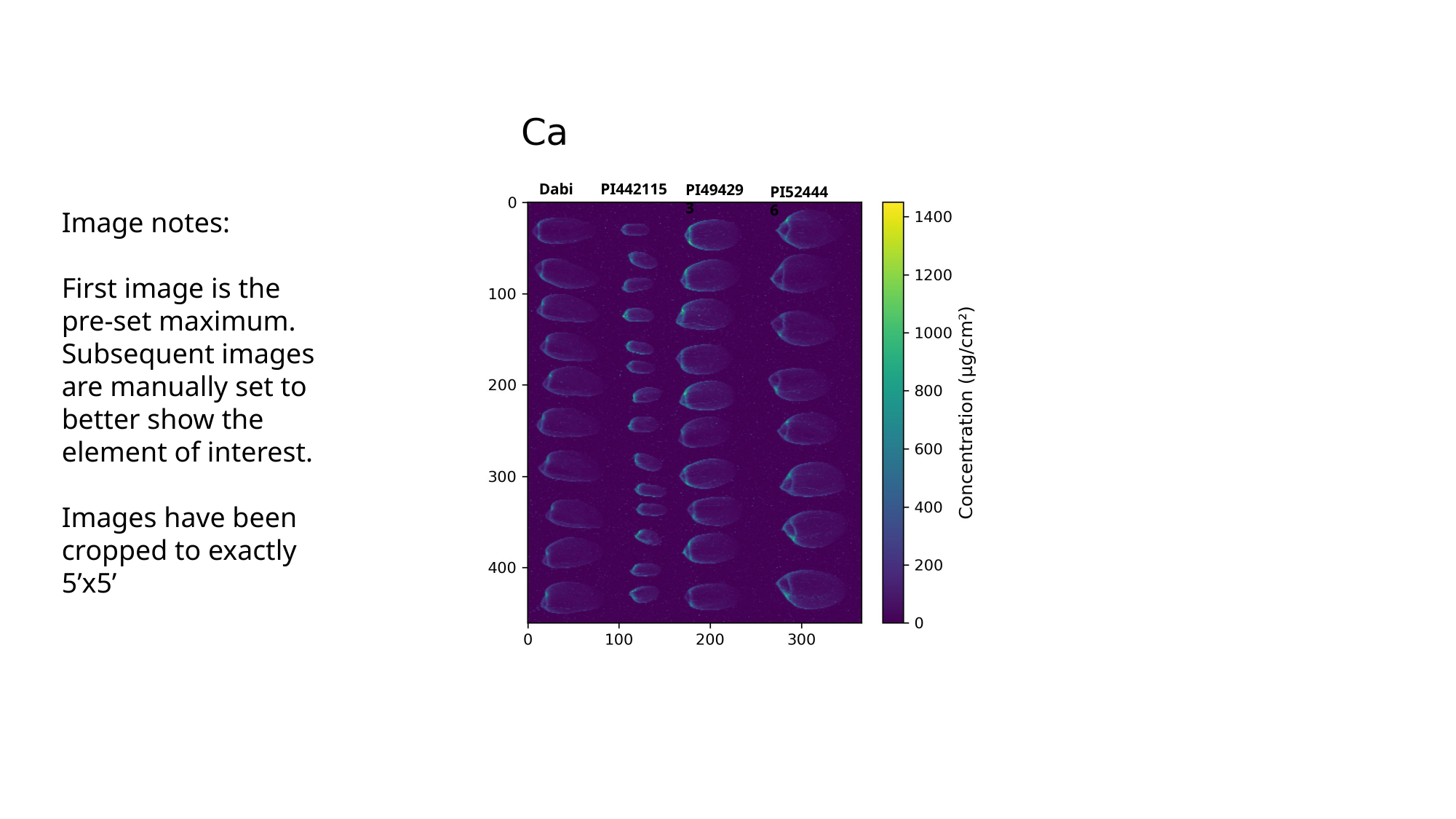

Dabi
PI442115
PI494293
PI524446
Image notes:
First image is the pre-set maximum.
Subsequent images are manually set to better show the element of interest.
Images have been cropped to exactly 5’x5’

## Slide 2
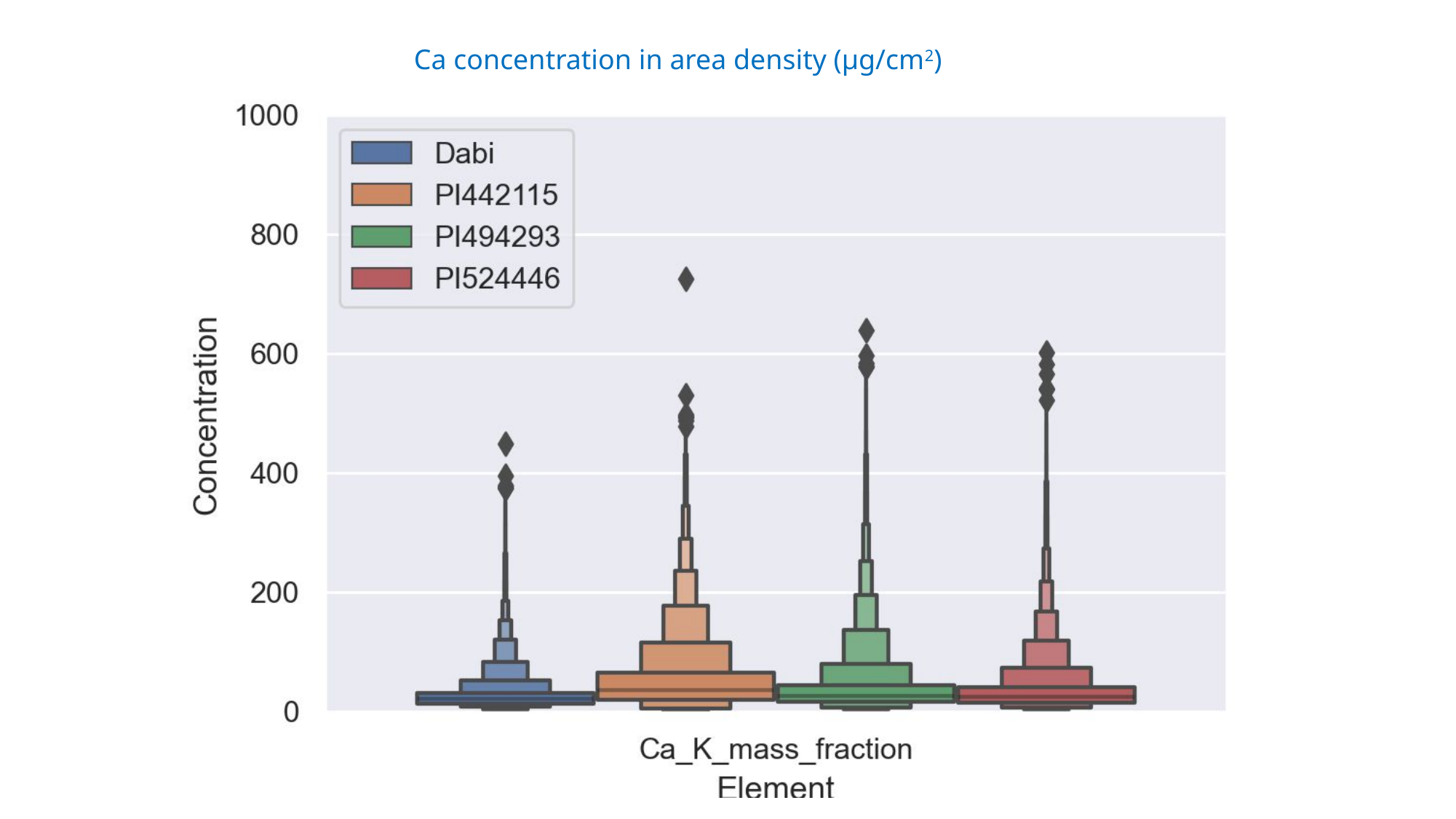

Ca concentration in area density (µg/cm2)

## Slide 3
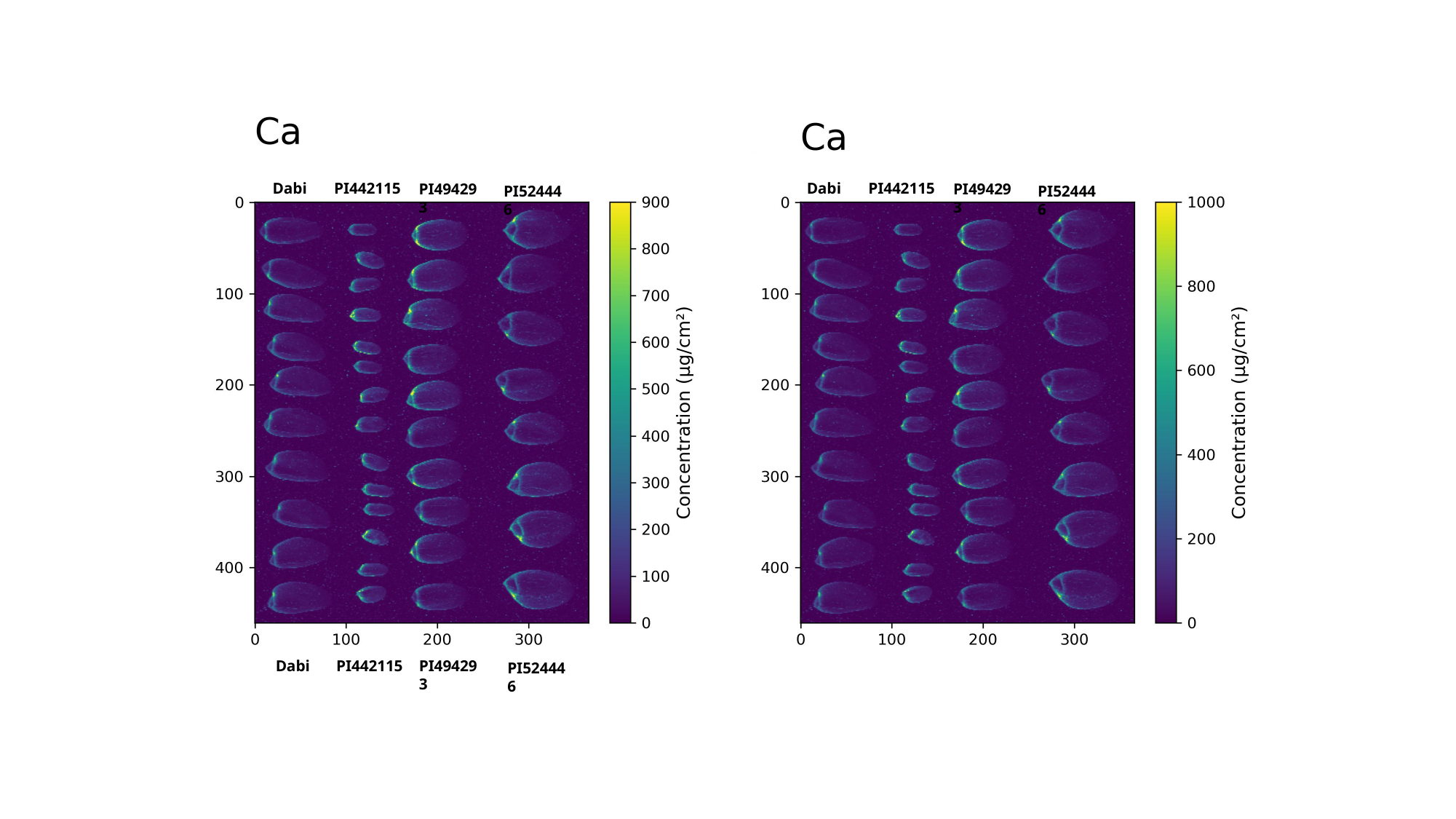

Dabi
Dabi
PI442115
PI442115
PI494293
PI494293
PI524446
PI524446
Dabi
PI494293
PI442115
PI524446

## Slide 4
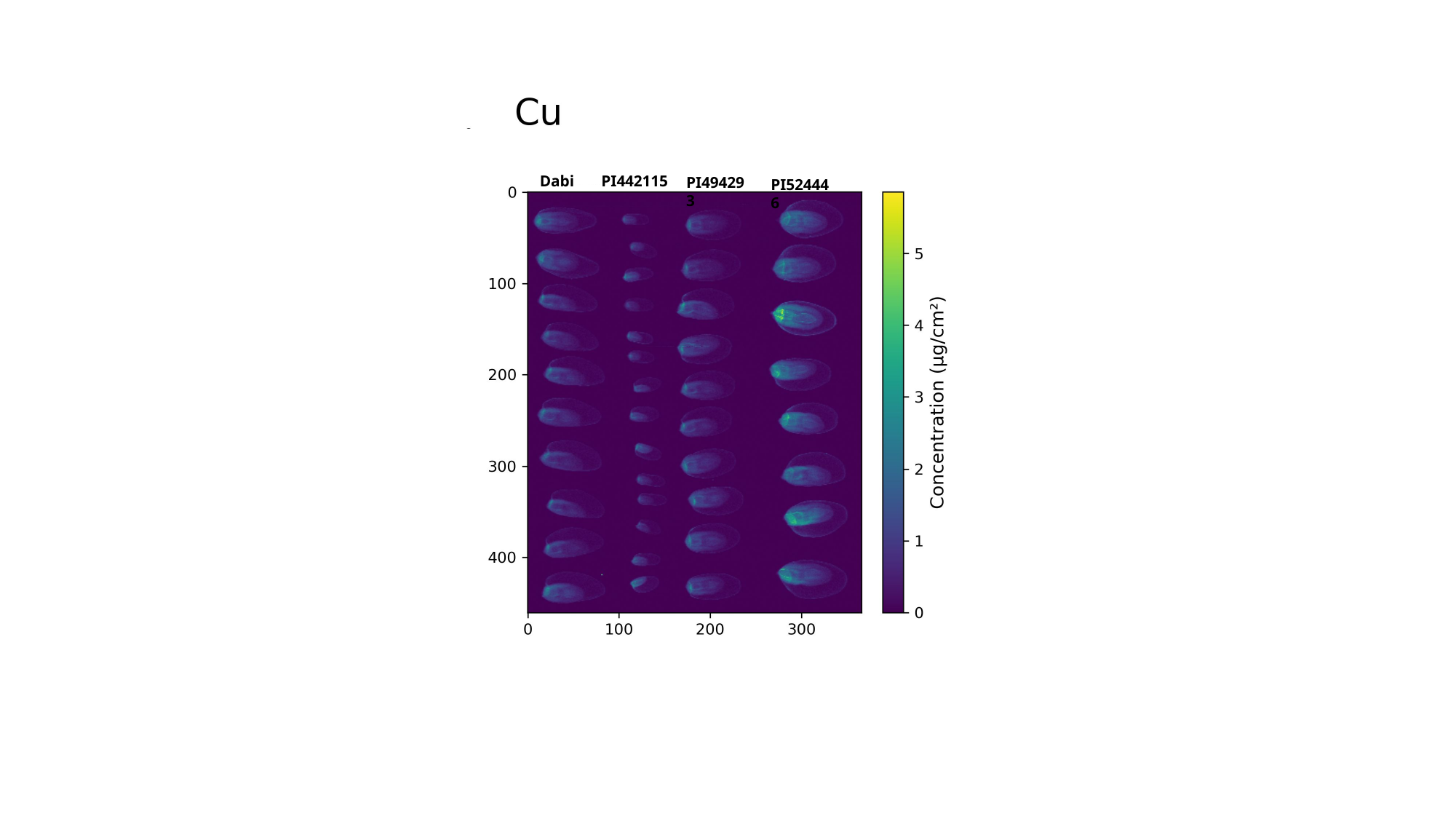

Dabi
PI442115
PI494293
PI524446

## Slide 5
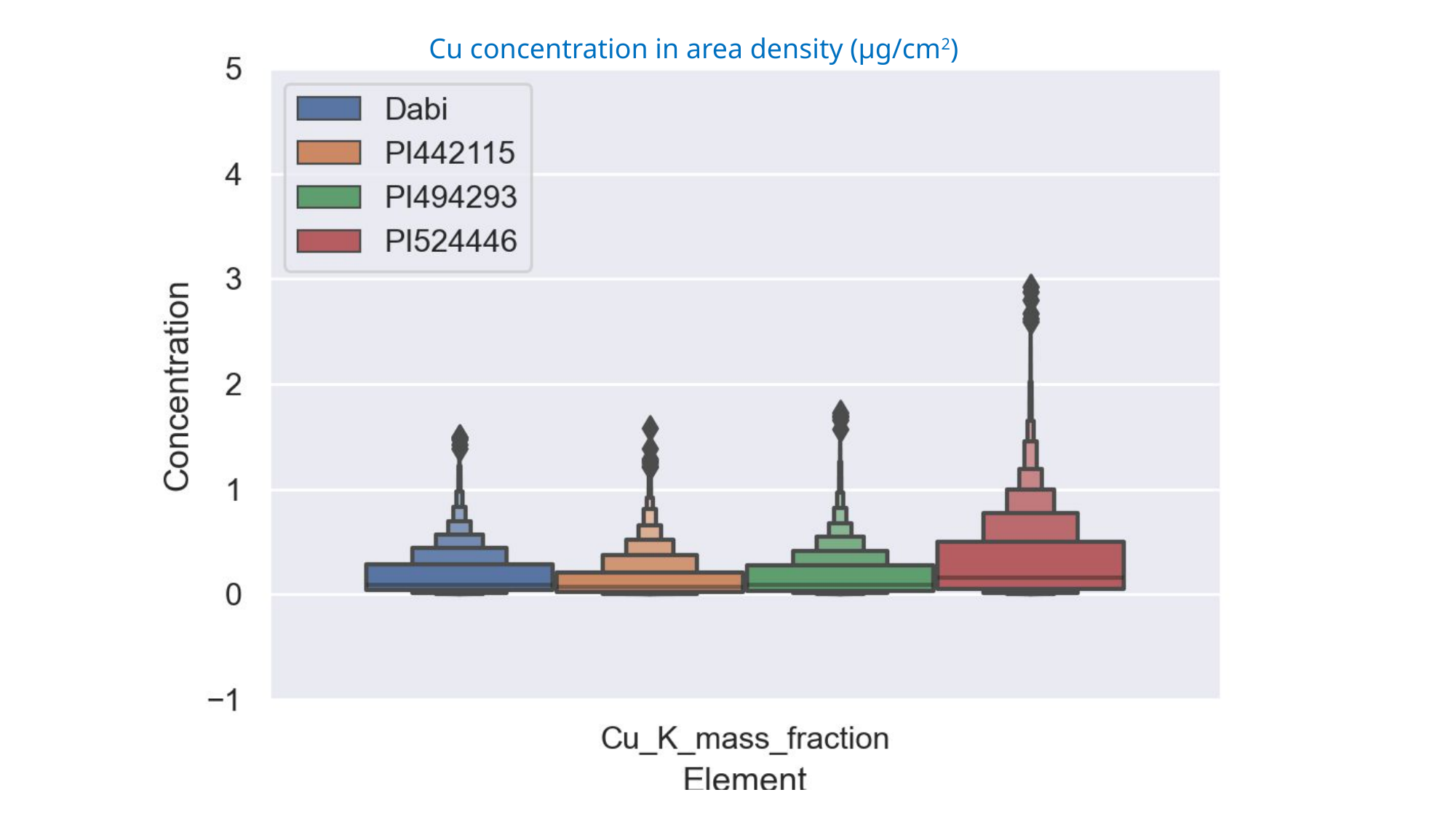

Cu concentration in area density (µg/cm2)

## Slide 6
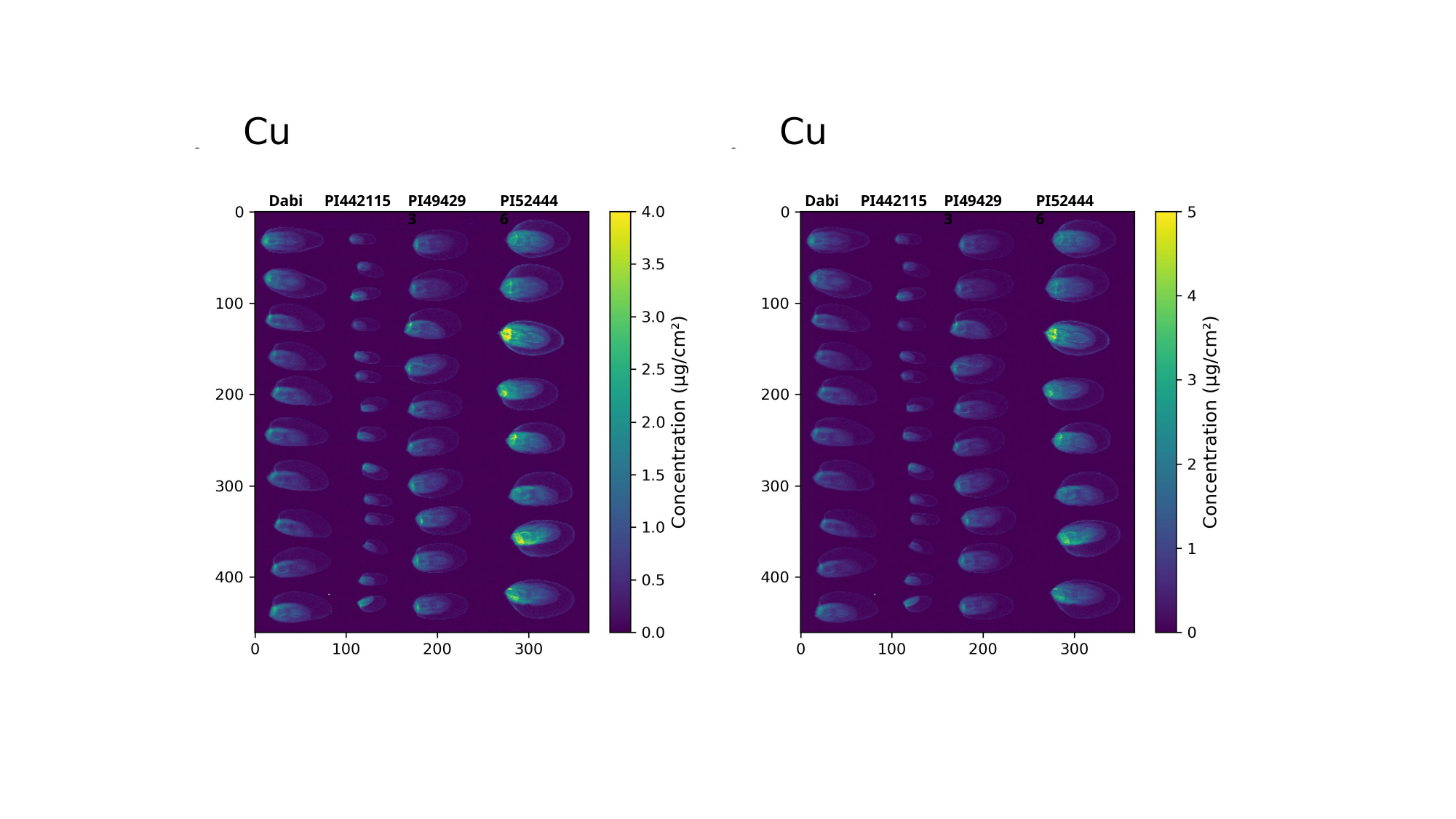

PI442115
PI442115
PI494293
PI524446
PI494293
PI524446
Dabi
Dabi

## Slide 7
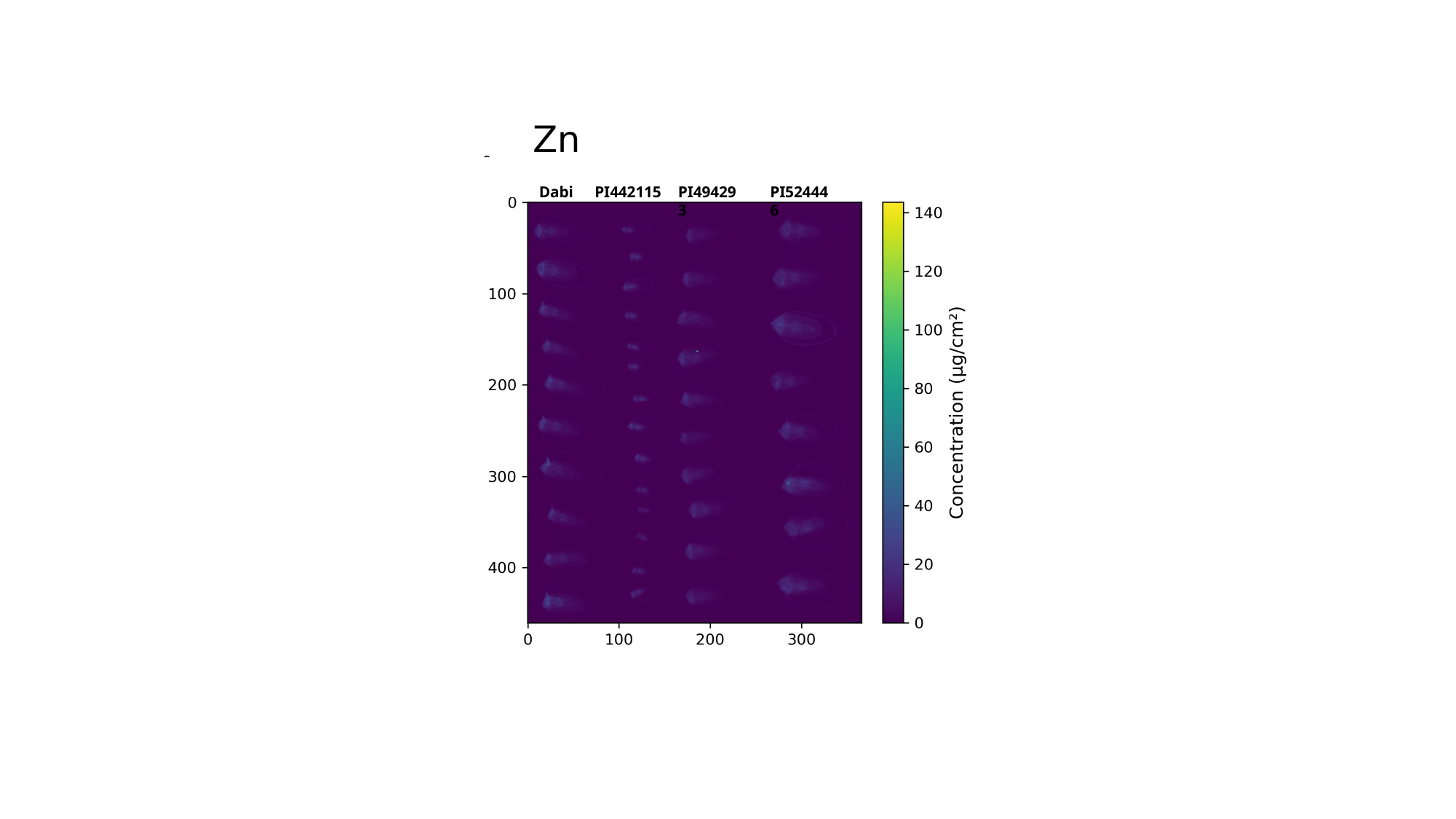

PI442115
PI494293
PI524446
Dabi

## Slide 8
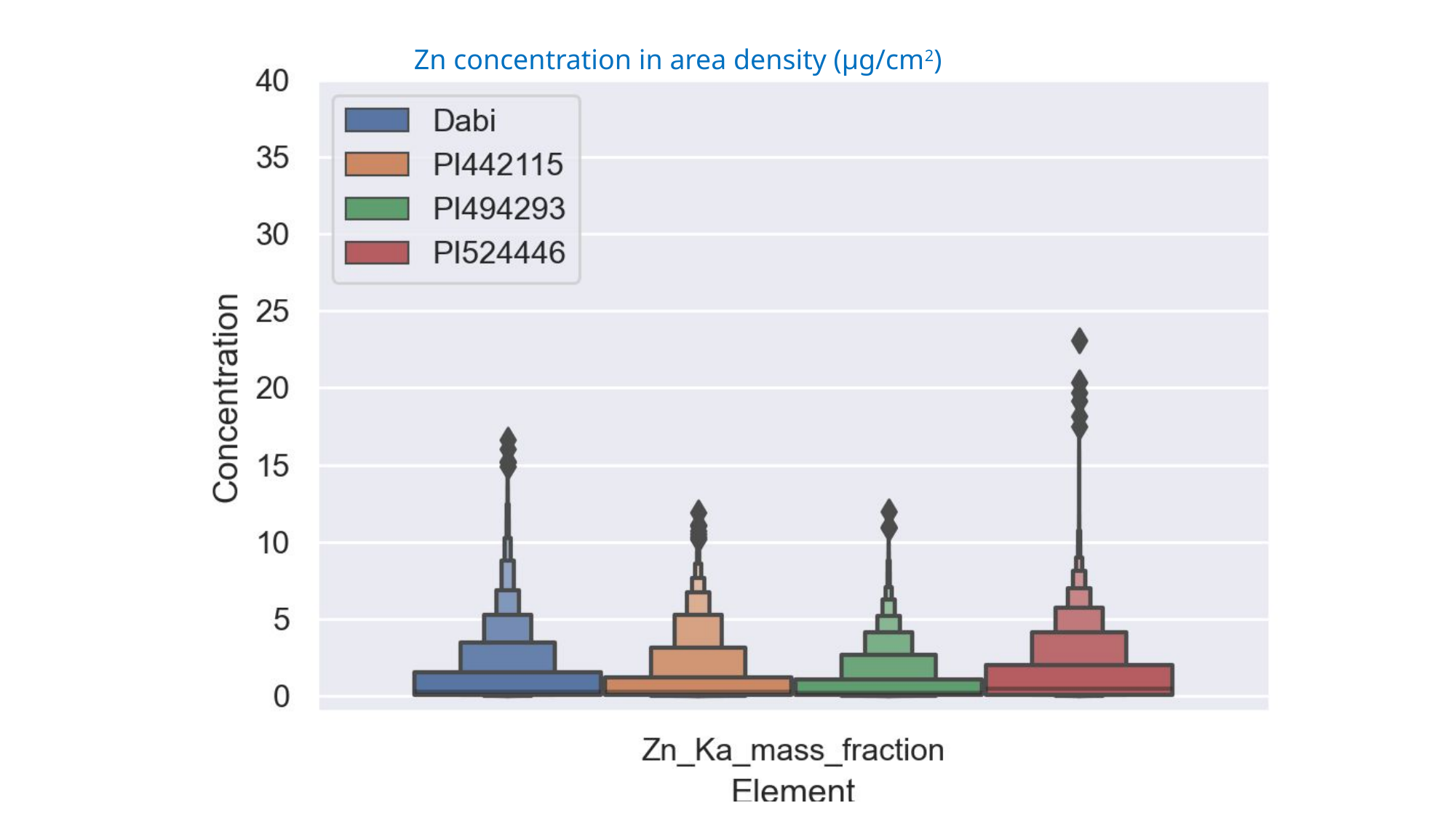

Zn concentration in area density (µg/cm2)

## Slide 9
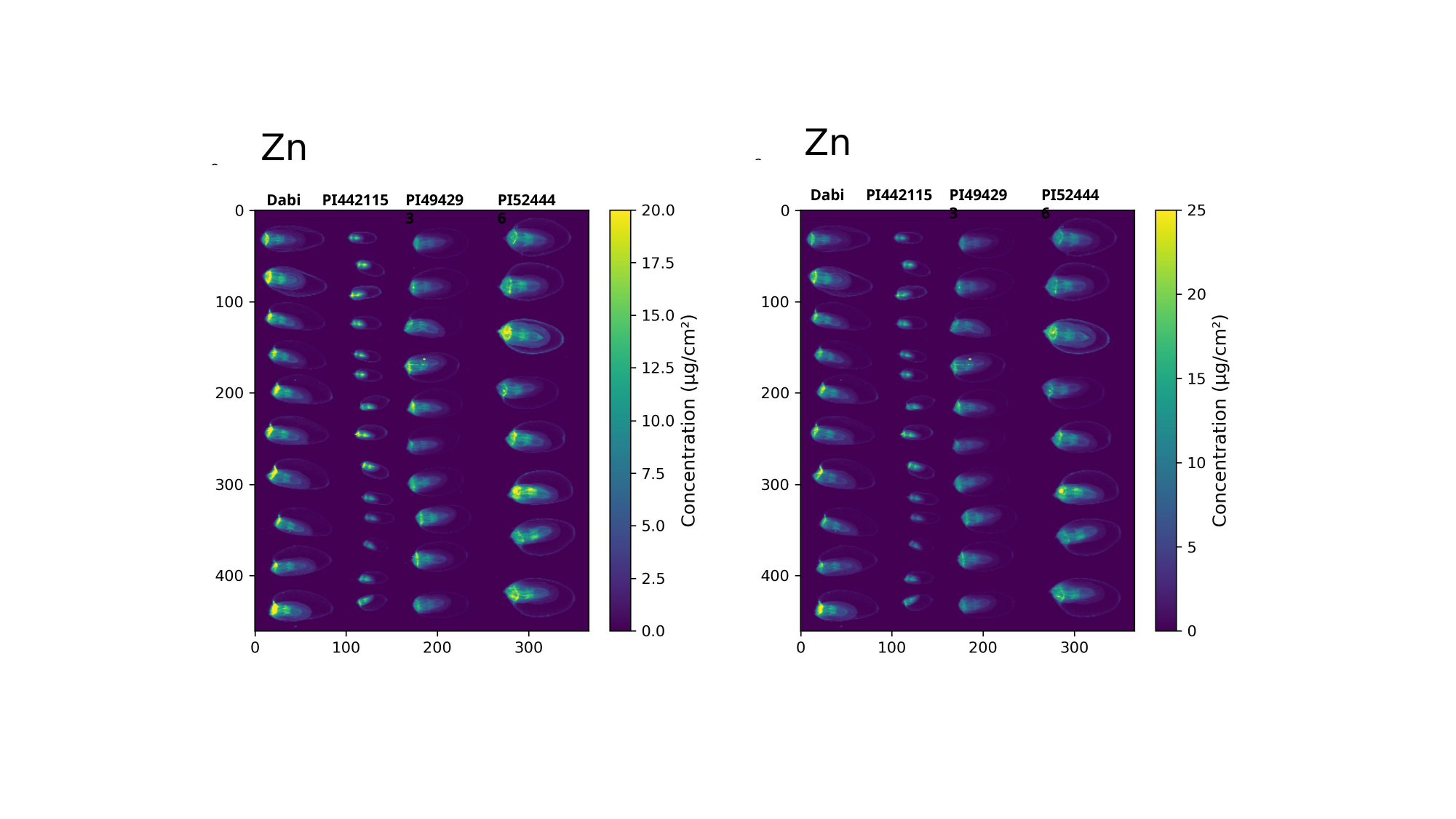

PI442115
PI494293
PI524446
Dabi
PI442115
PI494293
PI524446
Dabi

## Slide 10
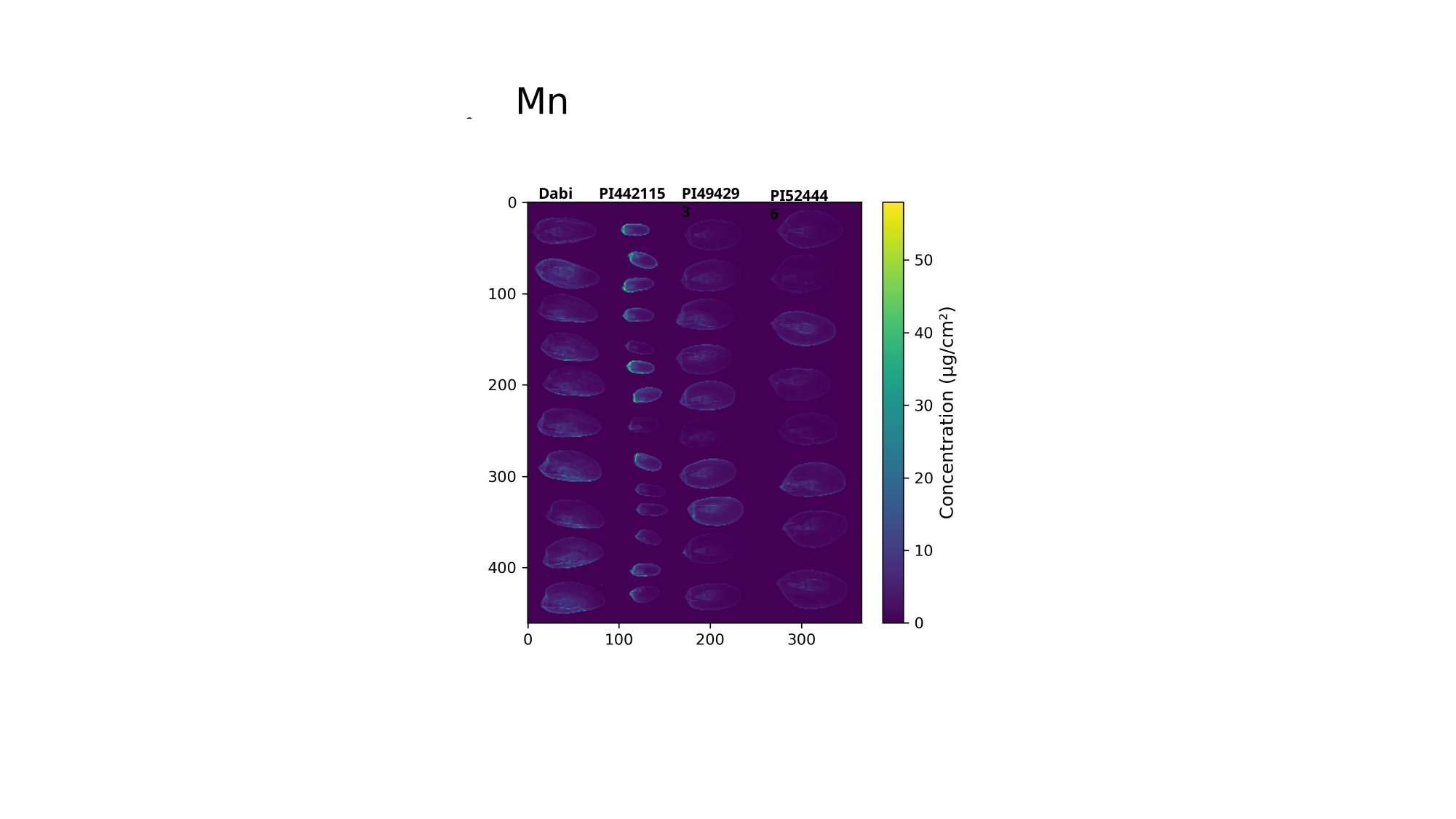

Dabi
PI494293
PI442115
PI524446

## Slide 11
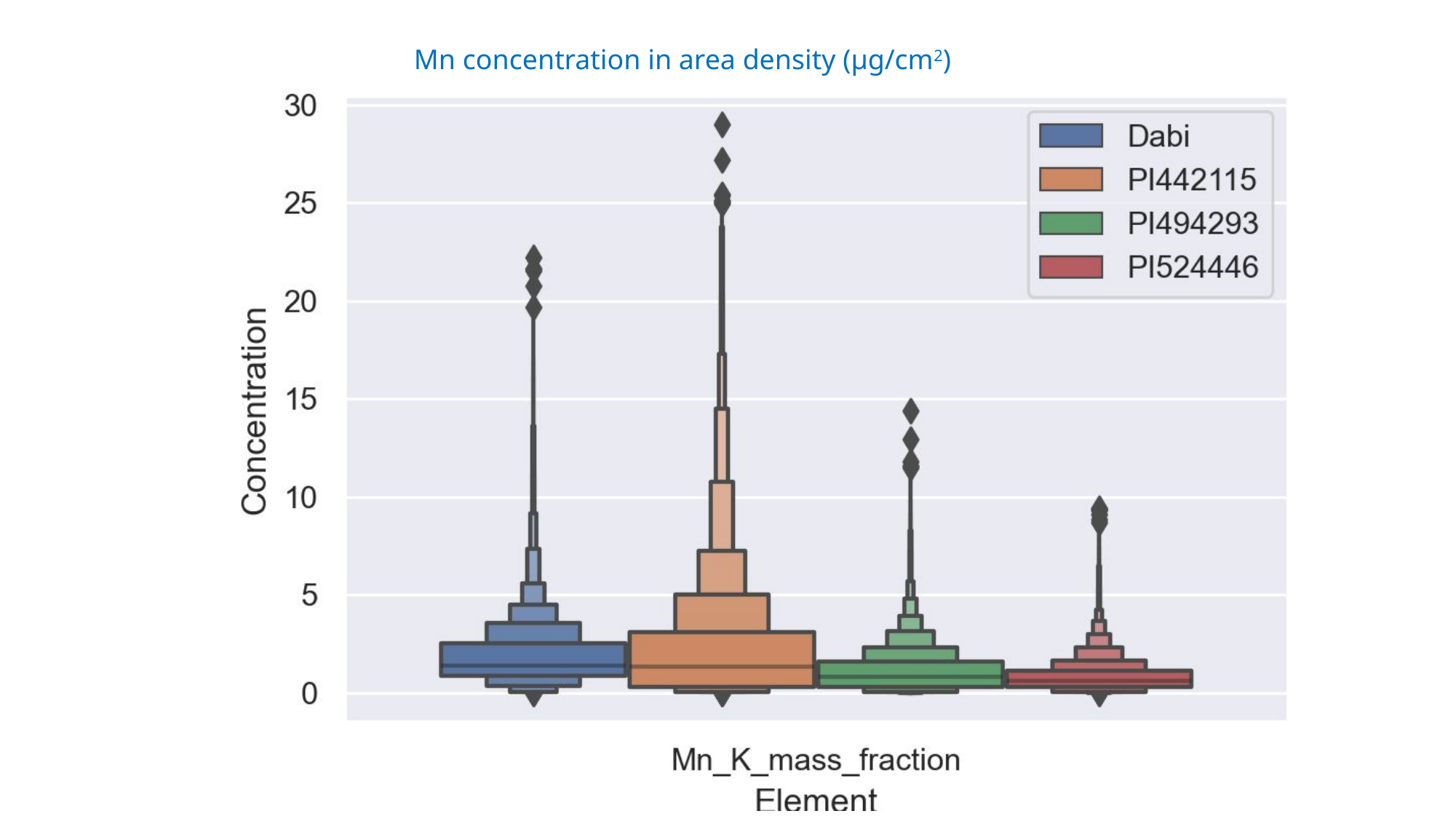

Mn concentration in area density (µg/cm2)

## Slide 12
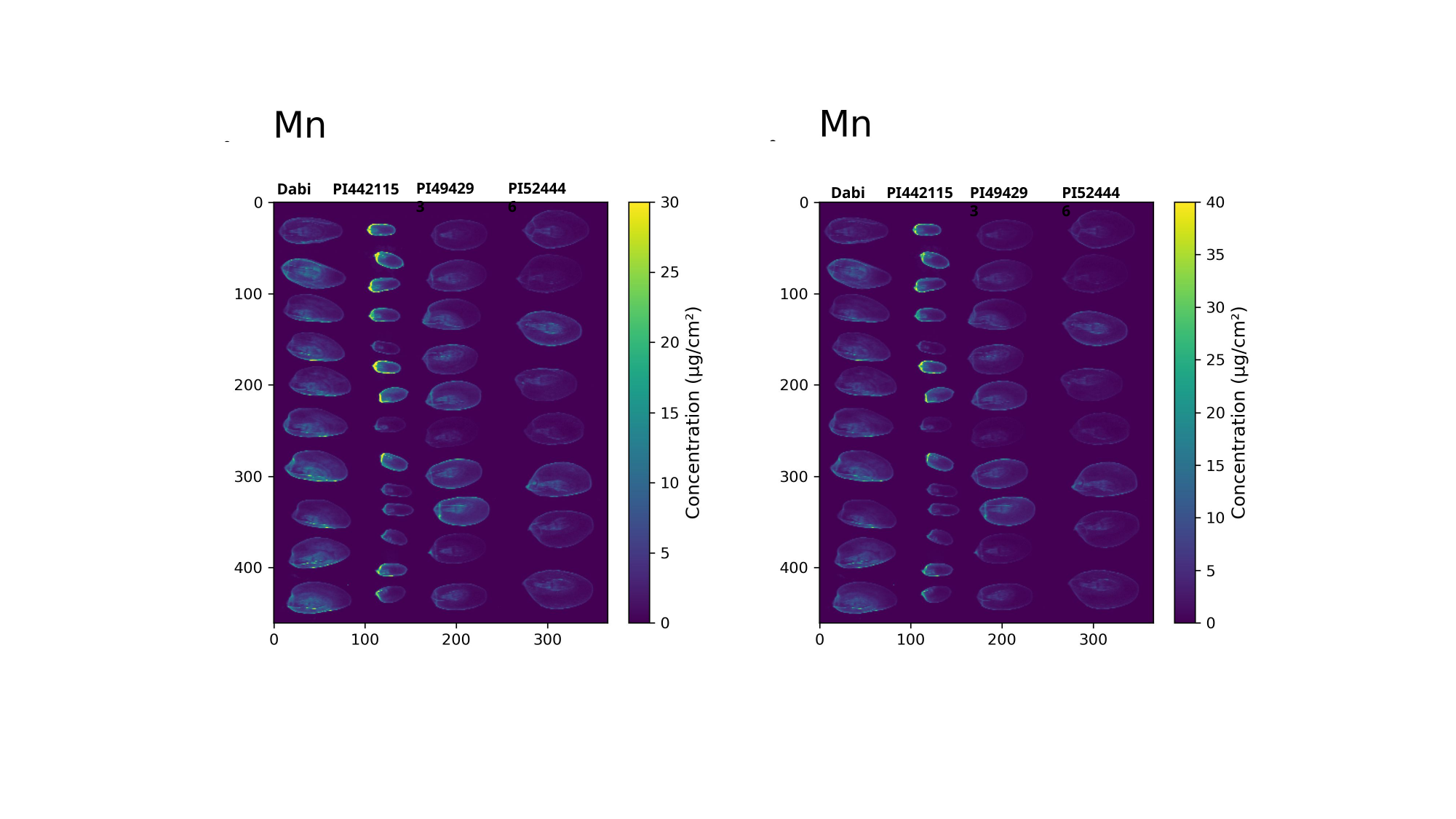

PI442115
PI494293
PI524446
Dabi
PI442115
PI494293
PI524446
Dabi

## Slide 13
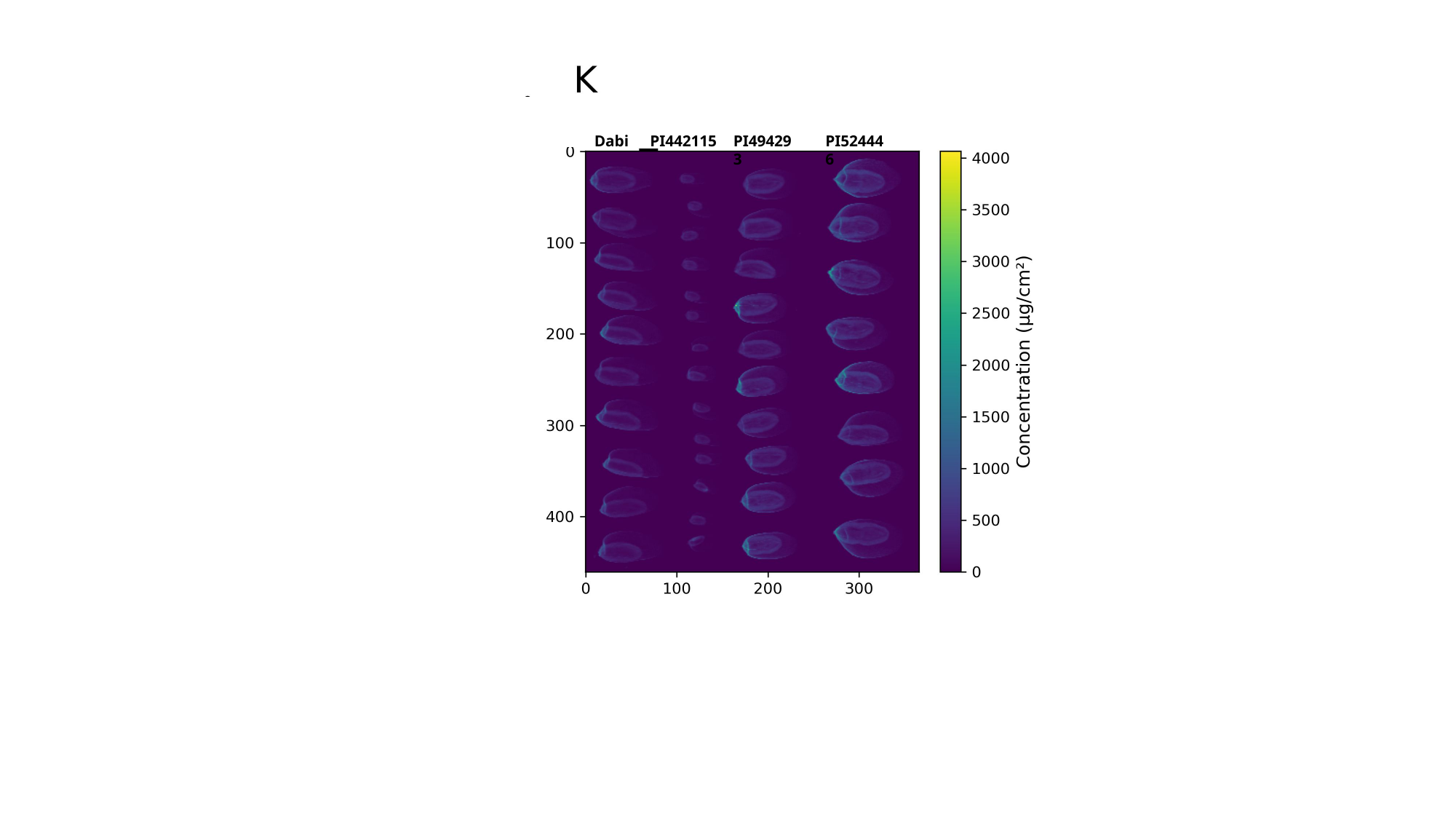

PI442115
PI494293
PI524446
Dabi

## Slide 14
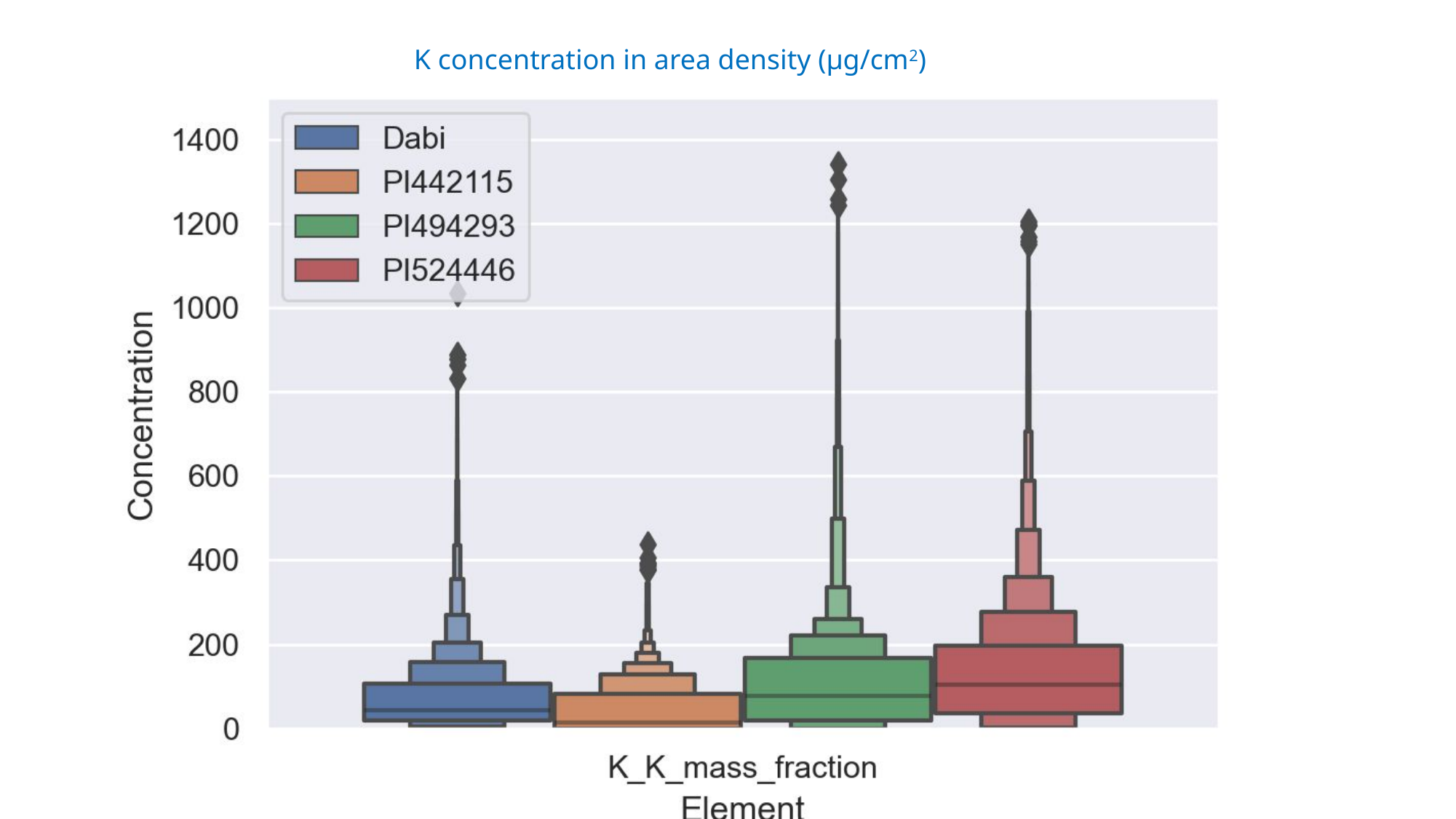

K concentration in area density (µg/cm2)

## Slide 15
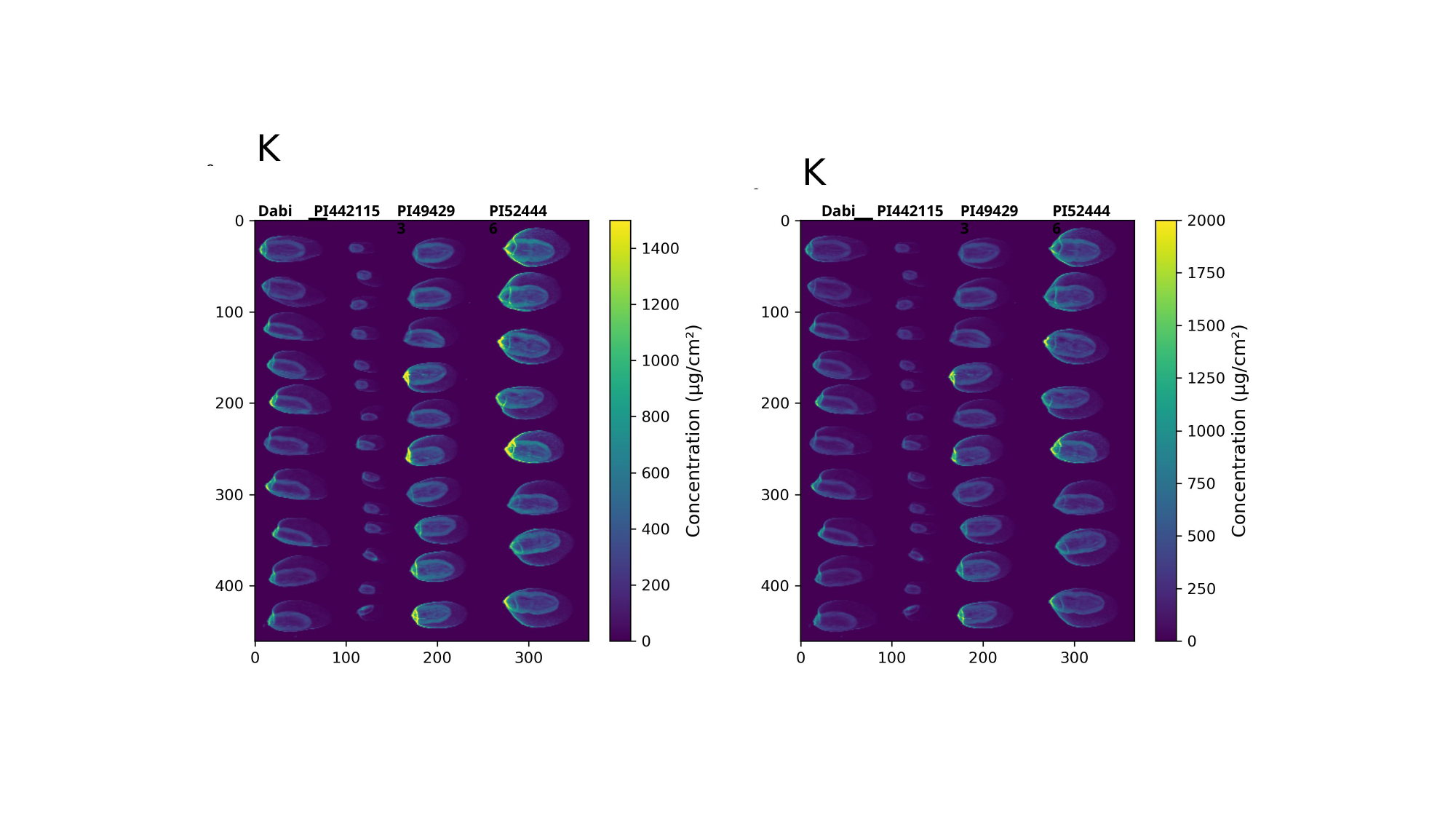

PI442115
PI494293
PI524446
PI442115
Dabi
PI494293
PI524446
Dabi

## Slide 16
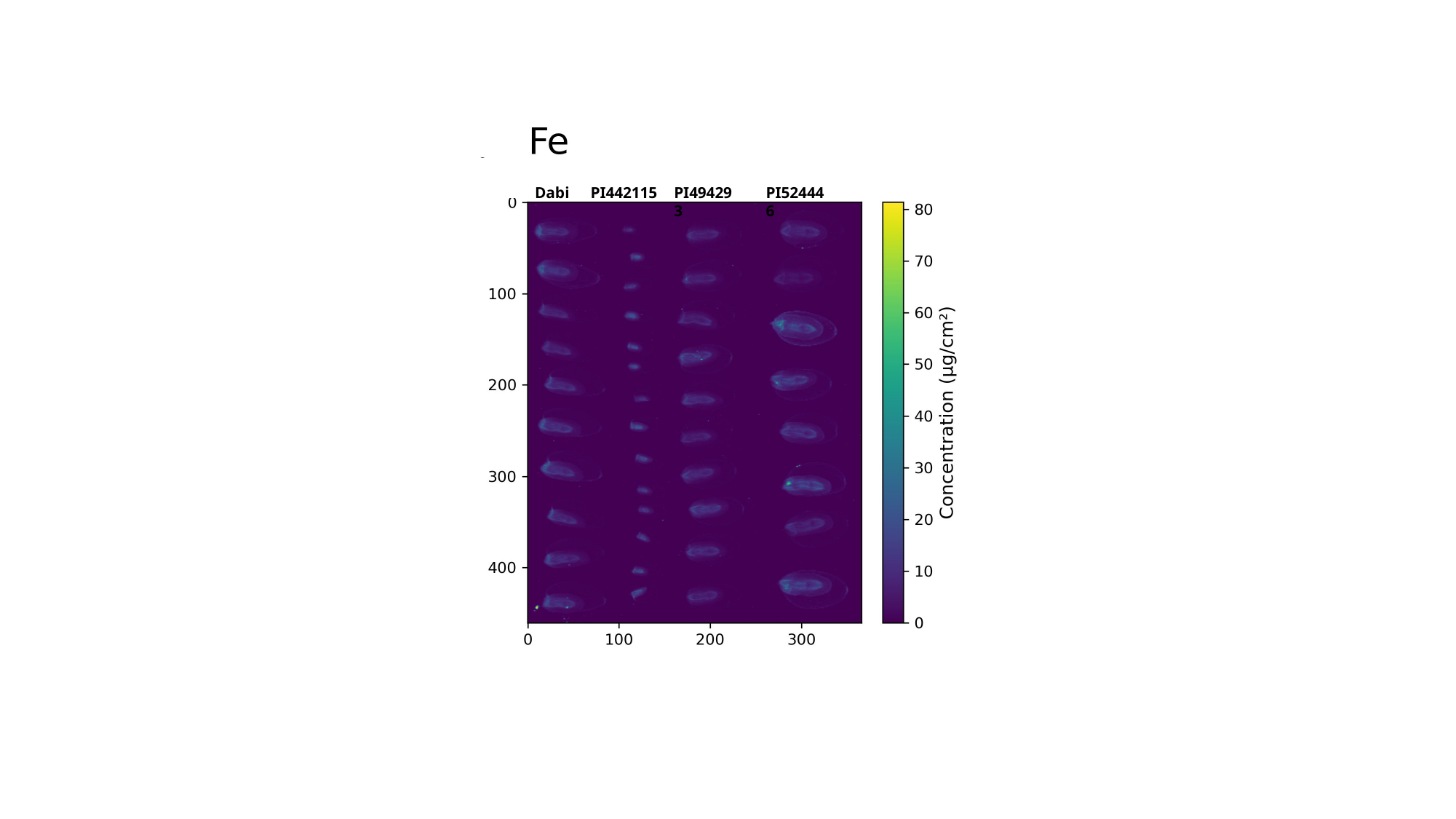

PI442115
PI494293
PI524446
Dabi

## Slide 17
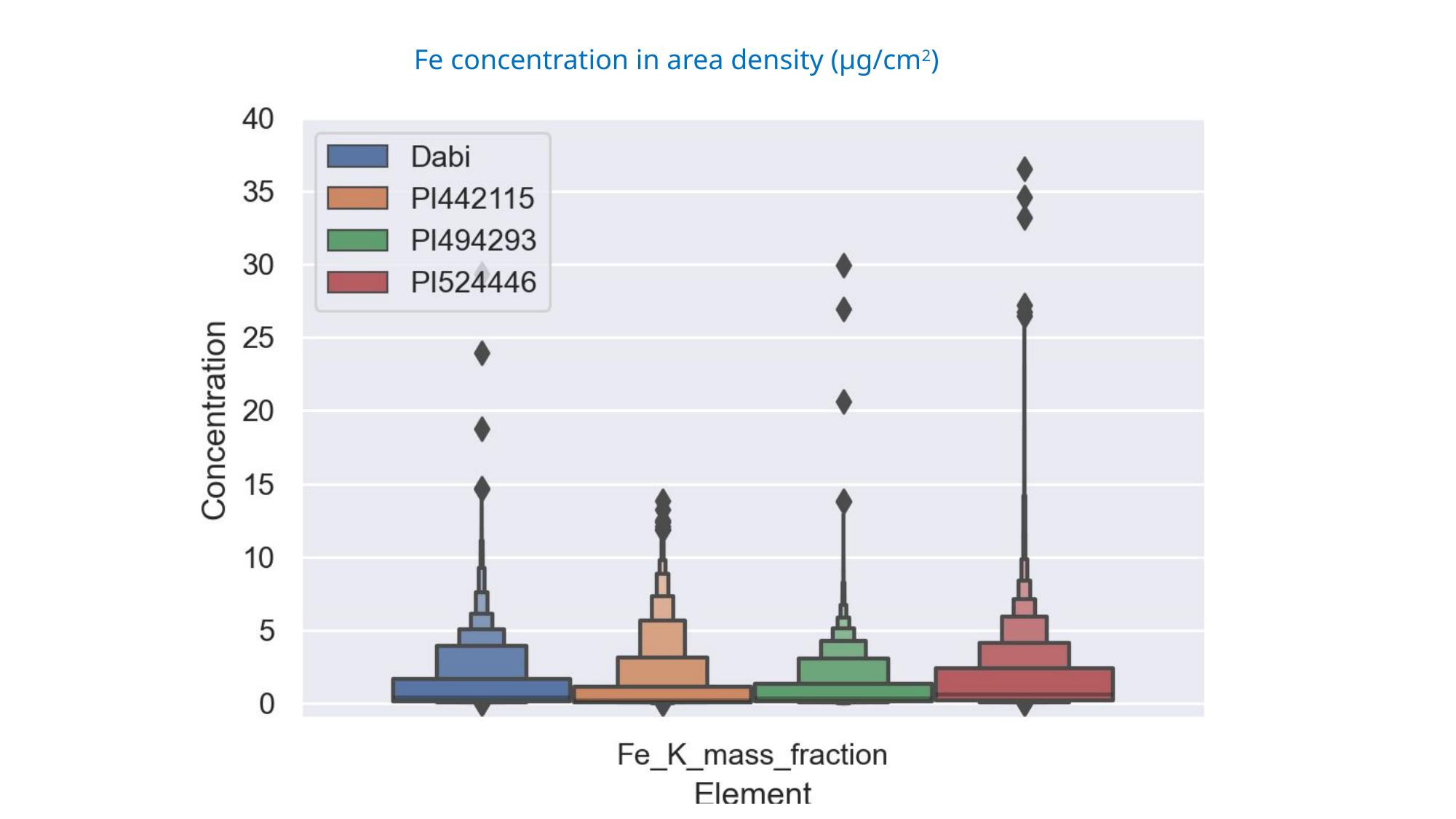

Fe concentration in area density (µg/cm2)

## Slide 18
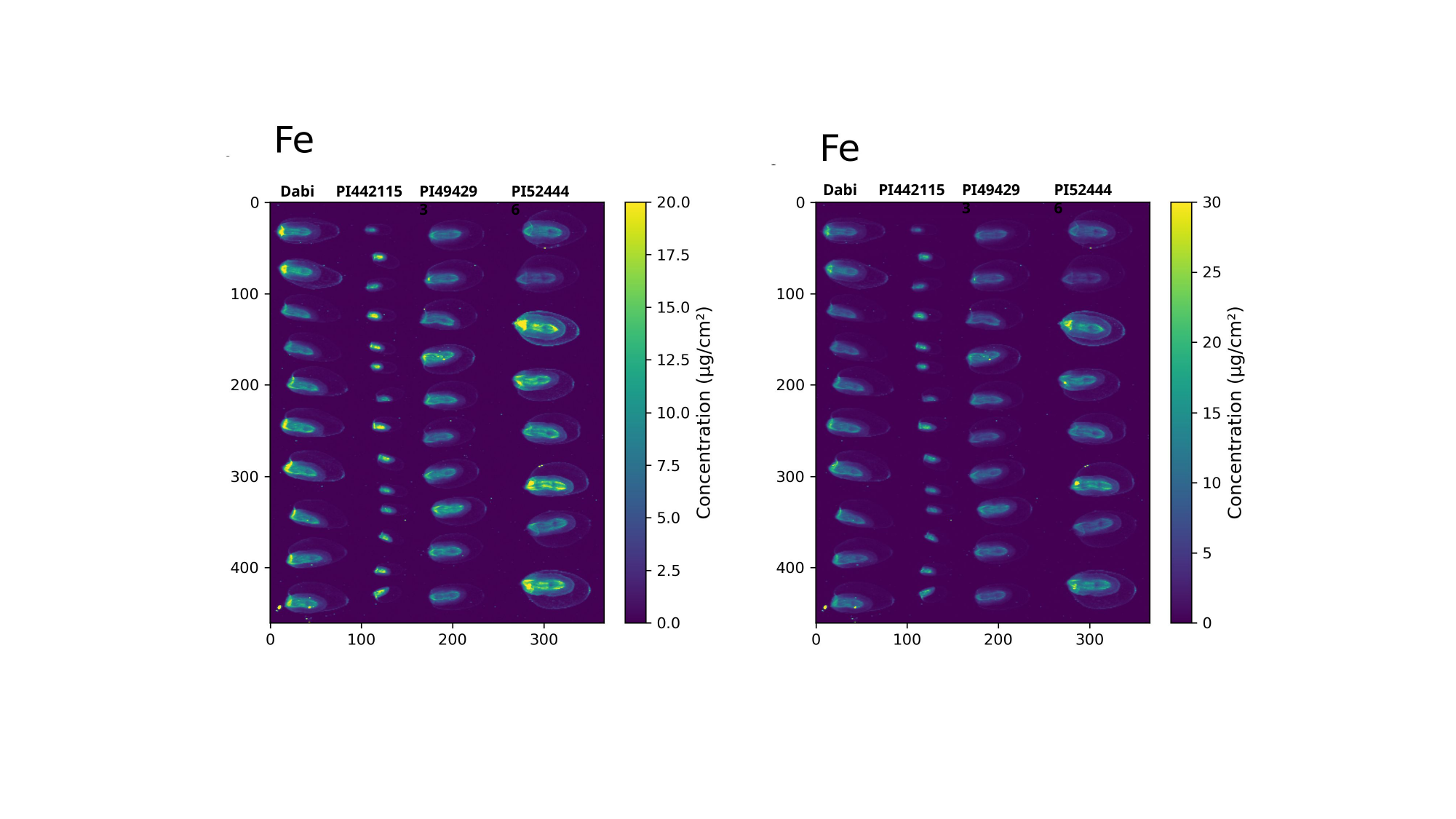

PI442115
PI494293
PI524446
Dabi
PI442115
PI494293
PI524446
Dabi
